# Eco-Friendly Synthesis of 1*H*-benzo[*d*]imidazole Derivatives by ZnO NPs Characterization, DFT Studies, Antioxidant and Insilico Studies

**DOI:** 10.3390/ph16070969

**Published:** 2023-07-06

**Authors:** Samar M. Mohammed, Wesam S. Shehab, Abdul-Hamid M. Emwas, Mariusz Jaremko, Magda H. Abdellattif, Wael A. Zordok, Eman S. Tantawy

**Affiliations:** 1 Department of Chemistry, Faculty of Science, Zagazig University, Zagazig 44519, Egypt; 2 Core Labs, King Abdullah University of Science and Technology (KAUST), Thuwal 23955-6900, Saudi Arabia; abdelhamid.emwas@kaust.edu.sa; 3 Biological and Environmental Science and Engineering (BESE), King Abdullah University of Science and Technology (KAUST), Thuwal 23955-6900, Saudi Arabia; mariusz.jaremko@kaust.edu.sa; 4 Department of Chemistry, Sciences College, Taif University, Taif 21944, Saudi Arabia

**Keywords:** 1*H*-benzo[*d*]imidazole, eco-friendly synthesis, nano ZnO, in silico studies DFT studies, antioxidant activity, molecular docking, pharmacokinetics

## Abstract

Benzimidazoles are classified as a category of heterocyclic compounds. Molecules having benzimidazole motifs show promising utility in organic and scientific studies. A series of mono-substituted benzimidazoles were synthesized by ZnO-NPs via cyclocondensation between substituted aromatic aldehydes and *o*-phenylene diamine. The synthesized compounds were characterized and compared with the traditional methods. The nano-catalyzed method displayed a higher yield, shorter time and recyclable catalyst. The DFT study and antioxidant activity were investigated for benzo[*d*]imidazole derivatives. Compound **2a** exhibited the highest antioxidant activity among the tested compounds. We focused on the catalytic activity of ZnO in the synthesis of heterocyclic structures with the goal of stimulating further progress in this field. The superiorities of this procedure are high yield of product, low amounts of catalyst and short reaction time.

## 1. Introduction

**Figure 1:**
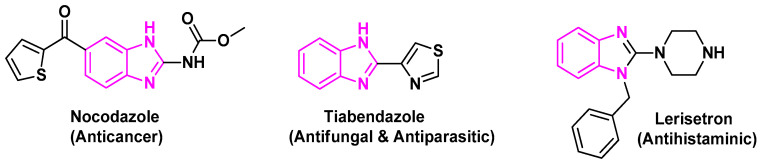
Benzimidazole core in some important drugs.

Benzimidazole or *1H*-1, 3-benzodiazole-based heterocycles are structurally much like and can evidently take the place of nucleotides, i.e., the adenine base of the DNA. This is regarded in addition to a factor of vitamin B. This feature has been substantially utilized in drug synthesis and medicinal chemistry, showing a huge variety of organic and scientific applications. Benzimidazole is also named 3-azaindole, benzimiinazole, benzoglyoxine. Benzimidazole is a vital modified structure that presents an extensive number of natural and pharmacologically active molecules. Magnetic nanoparticles (MNPs) have emerged as a new category of catalysts. This is referred to as ultrafine size and high surface area. Oxidation is a vital process in cell functionality and human life; imbalance in this biochemical process produces high amounts of free radicals like oxygen and nitrogen [1,2,3]. Oxidative stress of these free radicals leads to the damaging of proteins, DNA and other vital compounds in the body, which can cause chronic illness such as cancer, autoimmune disorders, cardiovascular diseases, diabetes, neurodegenerative diseases and aging [4,5,6]. Therefore, the needs of novel antioxidants have been the subject of intensive research. Heterocylic aromatic compounds with nitrogen atoms are considered as important pharmacophores owing to their versatile pharmacological activities [7] as anti-cancer [8,9], anti-oxidant [10], and anti-microbial [11]. Benzimidazoles are one of the most privileged heterocyclic substructures with wide biological activity, particularly as antioxidants [12,13]. The benzimidazole scaffold is found in many commercial drugs such as Nocodazole (anticancer), Tiabendazole (antifungal and antiparasitic), (Figure 1) [14,15]. The unique aspects of the benzimidazole core structure empower its ability of free-radical scavenging. Hence, the development of a nano-catalyzed environmentally benign protocol for the synthesis of benzimidazole derivatives is widely studied. Nano catalysts improve the yields and reduce the time of the reactions, in addition to their potential to be recycled many times [16,17]. In this regard, the high catalytic reactivity of ZnO nanoparticles and their environmental advantages make it an effective catalyst in organic reactions [18]. As a continuation of our work, some benzimidazoles have been synthesized using ZnO-NPs for their antioxidant examination and DFT studies.

In this article, we attempt to demonstrate the synthesis of some biologically important benzimidazole derivatives by ZnO NPs. This study covers conventional and new methods to synthesize certain pharmacologically active benzimidazole derivatives.

## 2. Results and Discussion

### 2.1. Chemistry

**Scheme 1:**
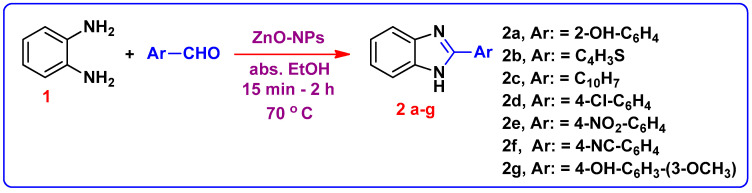
Synthetic route for formation of 1H-Benzimidazole derivatives **2**(**a**–**g**).

The reaction between *o*-phenylene diamine **1** and salicyaldehyde was selected for optimization of the reaction conditions. The reaction was carried out in the presence of two distinct nano catalysts (TiO_2_ and ZnO); the ZnO-NPs catalyzed reaction observed a greater yield. Accordingly, benzimidazoles **2b**–**2g** were achieved using ZnO-NPs as a catalyst (Scheme 1). Moreover, higher yields were obtained compared with the reported traditional approaches [19,20]. The data is summarized in Table 1. The structure of compound **2a** was confirmed by ^1^H-NMR spectrum by the presence of two singlet signals at 9.02 and 13.20 ppm assigned to OH and NH protons, respectively, and the aromatic protons displayed in the region of 6.52–7.43 ppm. Whereas, the ^13^C-NMR spectra shows a signal at (δ in ppm) 163.2 due to C=N-benzimidazole. For compounds **2b**–**2g**, their ^1^H NMR spectra showed NH signals appeared at 12.58–13.28 ppm.

**Scheme 2:**
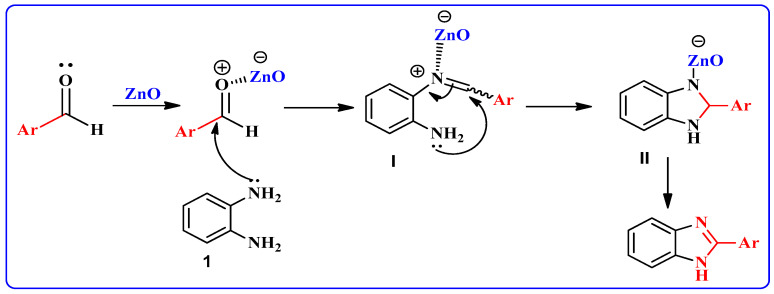
Possible reaction mechanism pathway for formation of 2-substituted benzimidazoles.

A possible mechanism for the formation of products in the presence of the nano-ZnO catalyst is shown in Scheme 2. At first, the aromatic aldehyde is activated by nano-ZnO. Intermediate **I** results by nucleophilic attack of *o*-phenylenediamine **1** to aromatic aldehyde. Subsequently, coordination of nitrogen to nano-ZnO facilitates intramolecular cyclization of intermediate **I** and the formation of intermediate **II**. Deprotonation of intermediate **II** results in the target product.

**Scheme 3:**
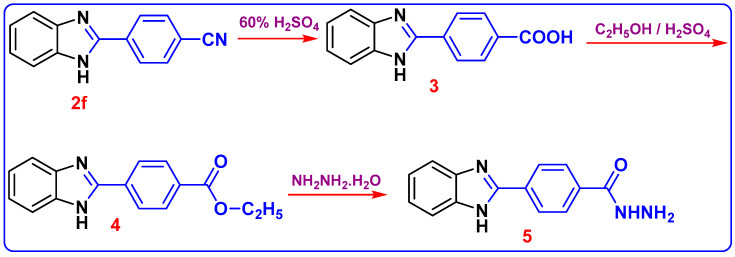
Synthesis of 4-(1*H*-benzoimidazol-2-yl)-benzoic acid hydrazide (5).

The chemical structure of compound **2f** was elucidated based on ^1^HNMR and ^13^C NMR analyses. The appearance of the shielded signal at 12.98 ppm corresponding to the NH group in the ^1^HNMR spectrum confirms the formation of 1*H*-benzimidazole moiety. Furthermore, the ^13^C NMR spectrum indicated the presence of SP and SP^2^ carbons attributed to C≡N and (C=N-benzimidazole) functions at (δ in ppm) 115.2 and 149.5. In Scheme 3, the compound **2f** further reacted with H_2_SO_4_ 60% to furnish 4-(1*H*-benzo[*d*]imidazol-2-yl) benzoic acid **3**. In its ^1^HNMR spectrum, the end cyclic NH and carboxylic OH protons resonated as two singlets at δ 13.19 and 10.25 ppm, respectively. The ^13^C NMR spectrum revealed the absence of C≡N carbon and presence of C=O of the carboxylic group at 167.0 ppm. The acid derivative **3** was then esterified in ethanol and a few drops of Conc. H_2_SO_4_ added to form 4-(1*H*-benzo[*d*]imidazol-2-yl)-benzoic acid ethyl ester **4**; the structure was confirmed with the spectroscopic analysis. In ^1^H NMR of compound **4**, triplet and quartet signals were revealed at 1.35 and 4.37 ppm, respectively, which was attributed to (OCH_2_CH_3_) ethyl ester protons. Other evidence of the formation of compound **4** is its ^13^C NMR, which accounted for 2 sp3 carbons at δ 14.61 and 61.65 for CH_2_ and CH_3_ ethyl protons, respectively. The C=O signal from the carbonyl of the ester group is also observed around 165.52 ppm. The carbon peak from the C=N group in the benzimidazole ring was also observed at 149.42 ppm as expected. The formation of hydrazide **5** was reached via hydrazonolysis of ethyl ester **4** with hydrazine hydrate in ethanol. The formation of **5** showed strong absorption bands at 3411, 3303, and 3158 cm^−1^ for the NH_2_/NH groups. The ^1^H NMR of **5** displayed the presence of singlet δ_H_ 12.52 for benzimidazole-NH and 4.35, 7.25 ppm for NH_2_, NH (hydrazide) proton; and the aromatic protons (Ar-H) were found in the spectrum at δ_H_ 7.56–7.72. ^13^C NMR (DMSO-*d*_6_) showed signals at δ_C_ 192.4 assigned to the C=O group, 140 ppm assigned to C-N, in addition to 109.5 ppm assigned to aromatic carbons at δ_C_ 134.7.

### 2.2. ZnO- NPs Examination

#### 2.2.1. XRD Examinations

**Figure 2:**
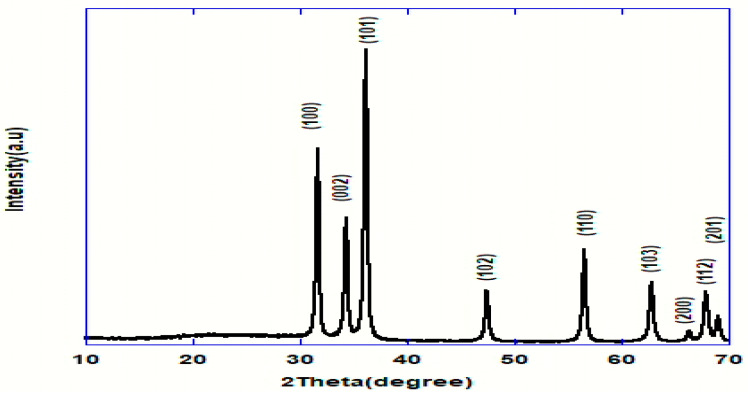
XRD pattern of ZnO nanoparticles.

Figure 2 signified the X-ray diffraction pattern of the ZnO-NPs between 2θ in the range of 10 and 70°. It demonstrated prominent peaks located at 2θ values of 31.84, 34.52, 36.33, 47.63, 56.71, 62.96, 68.13, and 69.18°, specified to the hexagonal ZnO structure, and coordinating in that order with (100), (002), (101), 102), (110), (103), (200), (112) and (201) reflection planes in agreement with the Joint Committee on Powder Diffraction Standards (JCPDS) card no 089-0510, which designates the formation of the monocrystalline phase [21,22]. As well, the lattice parameters of the hexagonal unit cell were proved to be almost (a = 3.249 Ǻ and c = 5.205 Ǻ), which is in good accordance with an earlier report [23].

#### 2.2.2. Morphological and Elemental Analyses (EDX)

**Figure 3:**
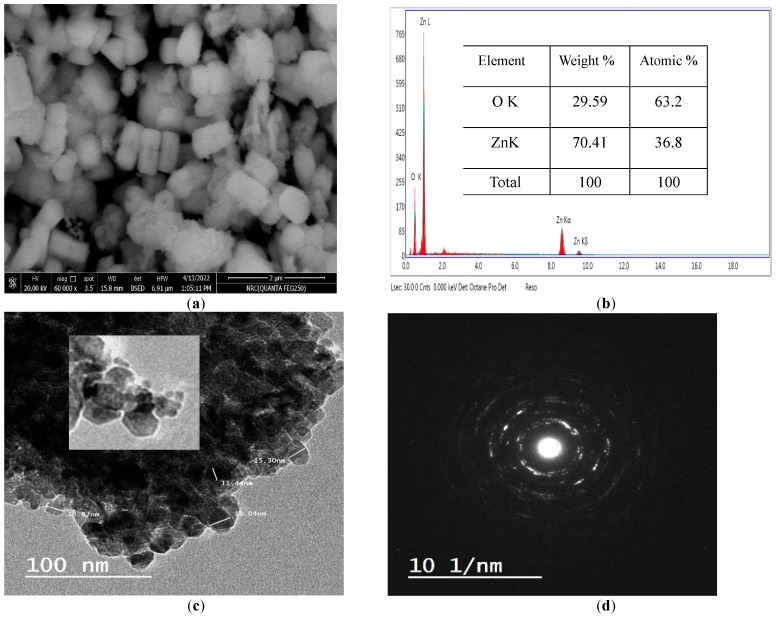
SEM (**a**), EDX (**b**), TEM (**c**) and SAED (**d**) of ZnO-NPs.

The morphological study revealed that the obtained ZnO-NPs seem to have uniform size in the form of arbitrarily distributed nanorods with a hexagonal (wurtzite) morphology. This complements the planes formed by its hexagonal structure as identified by XRD pattern. Particles had a plane surface in the nano size range [24,25] as revealed in Figure 3a. The remark of some larger nanoparticles in the SEM image was credited to the agglomeration of particles [26]. In addition, the rodlike structure is the best nanostructure compared to others; as a result of their one-dimensional nanostructures [27]. The EDX spectrum established the chemical composition of the ZnO-NPs. Figure 3b demonstrated the presence of only Zn and O elements; thus showing the purity of the prepared ZnO. No impurity was evident within the detection limit of the EDX. Therefore, it may be stated that the EDX proved that the synthesis process used was able to produce pure ZnO nanoparticles within its detection limit. TEM images presented in Figure 3c settled the hexagonal structure of the prepared ZnO nanoparticles as decided by the XRD [28]. The TEM picture Figure 3c together with the selected area electron diffraction (SAED) pattern seen in Figure 3d exhibited diagonally arranged concentric circles distinct with bright spots. The diffraction rings confirmed that the prepared ZnO was highly crystalline with the preferential orientation of nanocrystals [29,30]. As stated by Khan et al., the concentric rings may be acknowledged as the diffraction from the (100), (101), (102), (110), (103) and (112) plane of the polycrystalline structure (wurtzite) of the hexagonal crystal coordination [31].

#### 2.2.3. Zeta Potential

**Figure 4:**
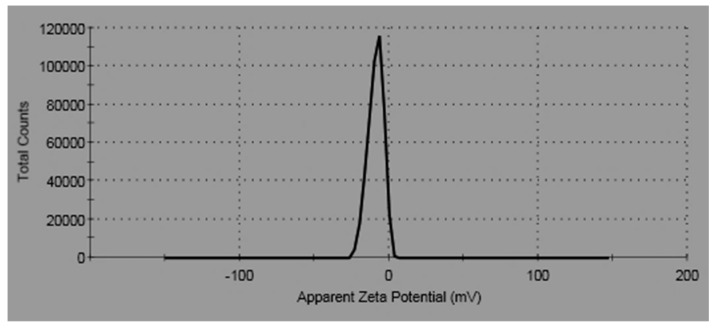
Zeta potential of ZnO-NPs.

The zeta potential (ξ) was used to recognize the surface charge of the particle, which performed as a sign of their colloidal stability. Nanoparticles with high positive or negative zeta potential display dispersion stability; as a result, they do not agglomerate on storage. The zeta potential of ZnO-NPs was found to be of negative value [−14.4 (mV)] as elucidated in Figure 4. The negative value is probably resultant to the samples’ negative charges. The negative zeta potential value designates that the dispersed ZnO NPs were crowned by negatively charged groups, which demonstrates their stability.

### 2.3. Antioxidant Evaluation

**Figure 5:**
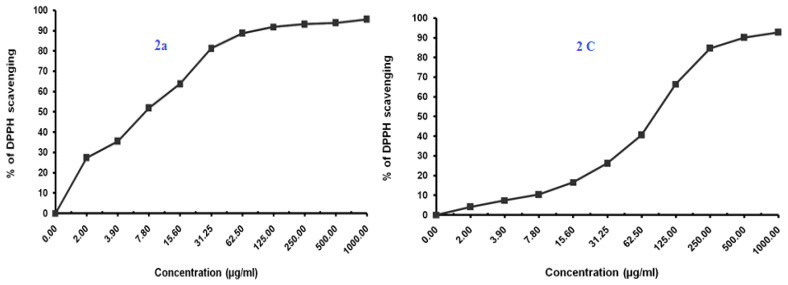

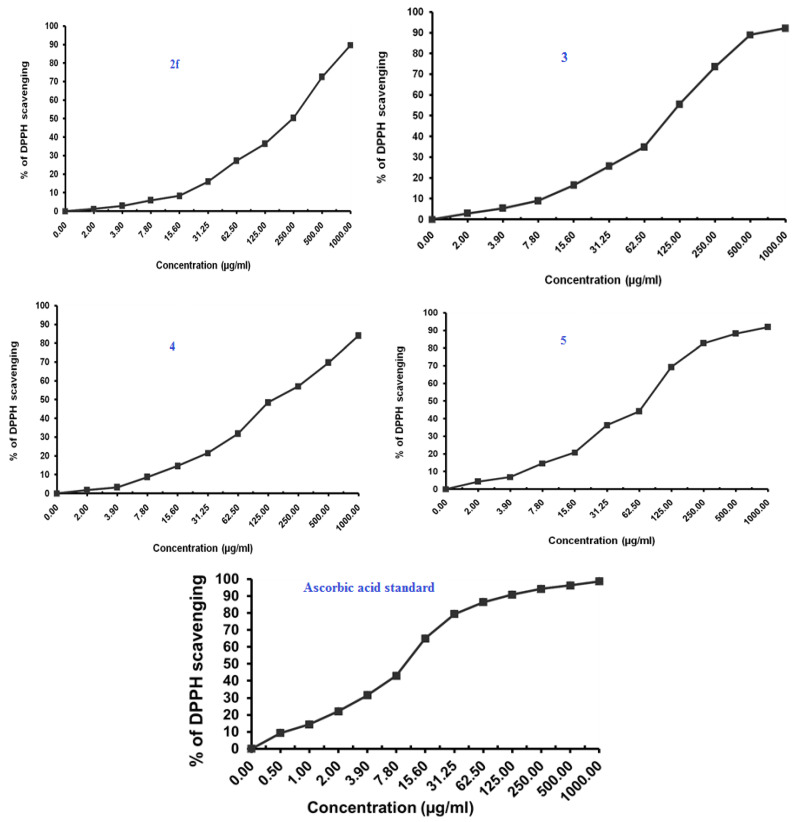
Antioxidant activity of benzimidazoles **2a**, **2c**, **2f**, **3**, **4** and **5** using DPPH Radical scavenging method.

The antioxidant activity was performed using the DPPH radical scavenging method wherein ascorbic acid was used as a positive control for comparison [32]. The results of the antioxidant activity of the compounds **2a**, **2c**, **2f**, **3**, **4** and **5** are shown in Table 2 and Figure 5.

The Compound **2a** at IC_50_ of 7.35 (µg/mL) seemed to be most active, which is assumed to be due to the presence of the free hydroxyl group on the aromatic ring, which is responsible for the antioxidant properties. The hydrogen from this group is donated to the free radical, resulting in a relatively stable free radical form. On the other hand compounds **2c** and **5** also exhibited moderate to significant antioxidant activity (77.07–85.21 µg/mL).

### 2.4. Insilico Studies

#### 2.4.1. Molecular Docking (Antioxidant)

The recently identified and generated drug targets were compared to ascorbic acid, a reference substance derived from the cytochrome c peroxidase enzyme (PDB code: 2X08), in the database’s molecular docking analysis to examine their potential as antioxidants. The goal of this research was to gain a better knowledge of how the chemicals created bind to the cytochrome c peroxidase enzyme’s protein-binding site.

**Figure 6:**
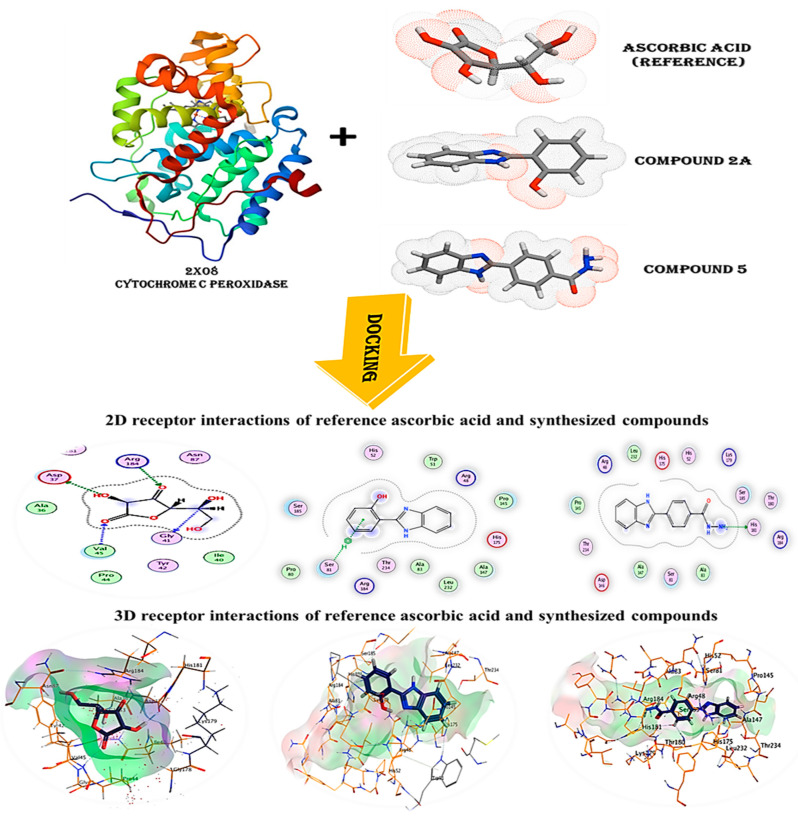
3D and 2D snapshots show the interaction of c peroxidase enzyme PDB: 2X08 with ascorbic acid and compounds (**2a** and **5**).

The co-crystallized ligand ascorbic acid was re-docked into the active site using the same number of criteria in order to validate the results of the current docking experiment at the active site. The root mean square deviation (RMSD) of the best-docked pose was 1.5912, and the energy score was −7.0409 kcal/mol, supporting the docking study performed with MOE software. Figure 6 shows that four hydrogen bonds were made between Asp37, Gly41, Val45 and Arg184 by ascorbic acid.

The predicted binding modes for compounds **2a** and **5** have energies of −7.3817 and −7.1462 kcal/mol, respectively. As seen in (Table 3), (Figure 6), compound **2a** successfully formed hydrogen bonds with Ser81, while compound **5** did the same with His181. The score of this molecule ensures that it will bind to the cytochrome c peroxidase enzyme’s protein-binding site with great stability.

#### 2.4.2. Pharmacokinetiks

##### Molinsipration

The synthesized substances **2a** and **5** expected pharmacokinetic/Molinspiration properties are listed in Table 4a. The majority of synthesized compounds showed promising bioactivity with the aid of Molinspiration virtual screening, as suggested by the docking parameters in Table 4b, which reveal the drug-like characteristics against kinase inhibitors, protease, and enzyme inhibitors. The calculated distribution of activity scores (version 2022.08), which include scores for around 100,000 typical drug-like compounds, are contrasted with scores for GPCR ligands, kinase inhibitors, ion channel modulators, nuclear receptor ligands, protease inhibitors and other enzyme targets. The score enables effective separation of molecules that are active and inactive.

**Figure 7:**
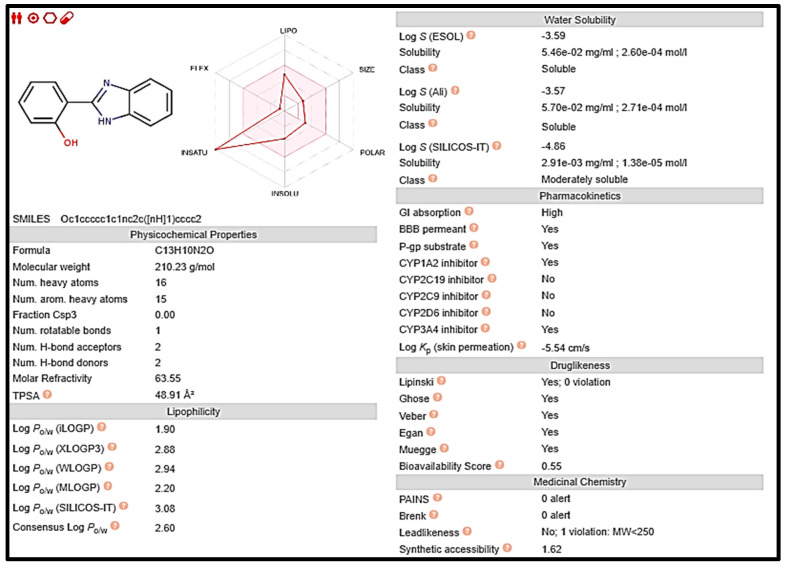
Computed values for prediction parameters of 2a.

**Figure 8:**
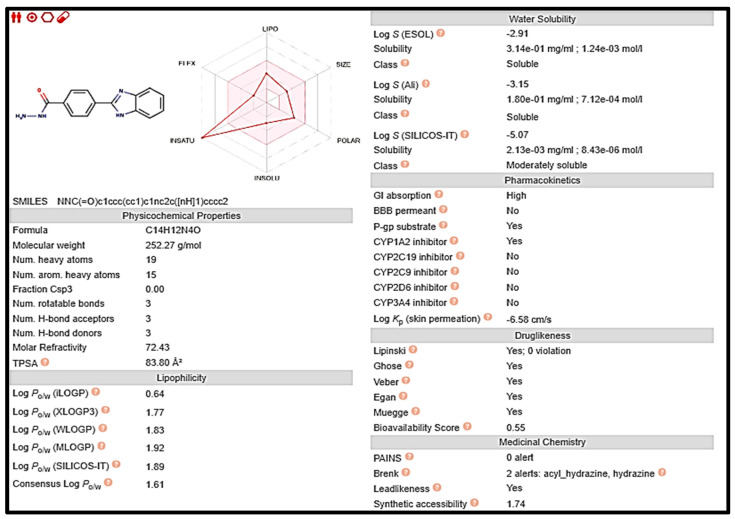
Computed values for prediction parameters of 5.

Drug-likeness and oral bioavailability analysis of compounds **2a** and **5** using Swiss ADME web re-sources:

Early on in the drug development process, it is crucial to analyze the pharmacokinetic features of candidate drugs. Lipinski and his team claim that drug-like **2a** and **5** must adhere to the rule of five (RO5), which is that they must have a molecular weight (MW) of 500 Da, 5 hydrogen bond donors (HBDs), and 10 hydrogen bond acceptors (HBAs). These requirements are depicted in Figure 7 and Figure 8, respectively [33].

## 3. Experimental

The melting points were measured by a digital Electrothermal IA 9100 Series apparatus Cole-Parmer, Beacon Road, Stone, Staffordshire, ST15 OSA, UK) and were uncorrected. IR spectra (KBrdiscs) were recorded on PyeUnicam Sp-3–300 or Shimadzu FTIR 8101 PC IR spectrophotometers (Cairo University, Cairo, Egypt). C, H and N analysis was carried out on a PerkinElmer CHN 2400. ^1^H and ^13^C NMR spectra were recorded on a Bruker 400 MHz NMR spectrometer using tetramethylsilane (TMS) as the internal standard; chemical shifts are expressed in *δ* (ppm), and DMSO-*d*_6_ was used as the solvent. All chemicals were purchased from Sigma-Aldrich (Taufkirchen, Germany), and all solvents were purchased from El-Nasr Pharmaceutical Chemicals Company (analytical reagent grade, Cairo, Egypt). All chemicals were used as supplied without further purification. The antioxidant activity was carried at the Regional Center for Mycology & Biotechnology (RCMB) Al-Azhar University, Naser City, and Cairo. The transmission electron microscopy (TEM) sample was loaded on a carbon-coated Cu grid (200 mesh) and examined at 200 KV using a JEM-2100 (JEOL, Tokyo, Japan) electron microscopy unit—Mansoura University.

### 3.1. General Procedure for the Synthesis of 2-Substituted 1H-Benzimidazoles ***2**(**a**–**g**)*

Benzaldehyde derivatives (10 mmol) and *o*-phenylenediamine (**1**) (10 mmol) were dissolved in absolute ethanol (50 mL) in the presence of (0.02 mol%) ZnO (nano particles). This mixture was stirred at 70 °C for 15 min–2 h. The product was washed repeatedly with an ethanol–water (1:1) mixture and then recrystallized from ethanol.

#### 3.1.1. 2-(1H-Benzo[d]imidazol-2-yl) Phenol (**2a**)

This compound was prepared by traditional methods [19,20]: yellow powder, yield (92%), m.p. = 226–228 °C. IR (KBr, cm^–1^): 3318 (OH, NH), 1591 (C=N). ^1^H-NMR: *δ*, ppm (DMSO-*d*_6_): 6.52 (t, 1H, ^3^J =7.00 Hz, Ar-H), 6.70 (d, 1H, ^3^J = 8.48 Hz, Ar-H), 7.23-7.43 (m, 4H, Ar-H), 9.02 (s, 1H, OH), 13.20 (s, 1H, NH). ^13^C NMR: *δ*, ppm (DMSO-*d*_6_): 113.51, 116.9, 119.8, 123.5, 127.7, 134.8, 136.7, 139.8, 163.2, 172.6 (C=N, imidazole and Ar-C). Anal. Calcd for C_13_H_10_N_2_O, (210.23):C, 74.27; H, 4.79; N, 13.33. Found: C, 74.25; H, 4.76; N, 13.34.

#### 3.1.2. 2-(Thiophen-2-yl)-1H-benzo[d]imidazole (**2b**)

This compound was prepared by traditional methods [34]: pale yellow powder, yield (53.3%), m.p. = 288–296 °C. IR (KBr, cm^–1^): 3320 (NH), 1591 (C=N); ^1^H-NMR: δ, ppm (DMSO-*d*_6_): 7.83 (d, 1H, Ar-H), 7.73 (d, 1H, Ar-H), 7.61 (d, 1H, Ar-H), 7.50 (d, 1H, Ar-H). 7.16–7.24 (m, 3H, Ar-H), 12.90 (s, 1H, NH). Anal. Calcd for C_11_H_8_N_2_S (200.26): C, 65.97; H, 4.03; N, 13.99. Found: C, 74.25; H, 4.76; N, 13.34.

#### 3.1.3. 2-(Naphthalen-1-yl)-1H-benzo[d]imidazole (**2c**)

This compound was prepared by traditional methods [34]: Pale brown powder, Yield (61.8%), m.p. = 224–226 °C. IR (KBr, cm^–1^): 3323 (NH), 1591 (C=N). ^1^H-NMR: δ, ppm (DMSO-*d*_6_): 7.23–8.11 (m, 10H, Ar-H), 9.11 (d, 1H, ^3^J = 8.16 Hz, Ar-H), 12.93 (s, 1H, NH). ^13^C-NMR: *δ*, ppm (DMSO-*d*_6_): 111.8, 119.5, 122.0, 123.3, 125.7, 126.8, 127.7, 1283, 130.6, 131.9, 132.3, 133.6, 134.9, 135.8, 143.2, 144.3, 151.2 (C=N, benzimidazole). Anal. Calcd for C_17_H_12_N_2_ (244.29): C, 83.58; H, 4.95; N, 11.47. Found: C, 83.55; H, 4.97; N, 11.45.

#### 3.1.4. 2-(4-Chlorophenyl)-1H-benzo[d]imidazole (**2d**)

This compound was prepared by traditional methods [20,33,34]: Pale brown powder, Yield (61%), m.p. = 296–298 °C. IR (KBr, cm^–1^): 3318 (NH), 1490 (C=N). ^1^H-NMR: δ, ppm (DMSO-*d*_6_): 7.20 (d, 2H, ^3^J = 8.48 Hz, Ar-H), 7.58–7.64 (m, 4H, Ar-H), 8.17 (d, 2H, ^3^J = 8.70 Hz, Ar-H), 12.97 (s, 1H, NH). ^13^CNMR: *δ*, ppm (DMSO-*d*_6_): 107.7, 116.5, 116.7, 128.0, 129.3, 130.3, 130.4, 130.9, 131.6, 133.2, 160.9 (Ar-C). Anal. Calcd for C_13_H_9_ClN_2_ (228.7): C, 68.28; H, 3.97; N, 12.25 Found: C, 68.30; H, 3.95; N, 12.20.

#### 3.1.5. 2-(4-Nitrophenyl)-1H-benzo[d]imidazole (**2e**)

This compound was prepared by traditional methods [35,36]: Brown powder, Yield (57.3%), m.p. = 320–322 °C. IR (KBr, CM^−1^): 3366 (NH), 1593 (C=N). ^1^H-NMR: δ, ppm (DMSO-*d*_6_): 7.26 (d, 2H, ^3^J = 7.9 Hz, Ar-H), 7.59–7.71 (m, 4H, Ar-H), 8.40 (d, 2H, ^3^J = 8.2 Hz, Ar-H), 13.28 (s, 1H, NH). ^13^CNMR: δ, ppm (DMSO-*d*_6_): 112.4, 119.4, 122.3, 124.0, 124.2, 127.3, 130.5, 136.0, 143.8, 147.7, 148.9 (C=N, imidazole and Ar-C). Anal. Calcd for C_13_H_9_N_3_O_2_ (239.2): C, 65.27; H, 3.79; N, 17.56. Found: C, 65.24; H, 3.77; N, 17.60.

#### 3.1.6. 4-(1H-benzo[d]imidazol-2-yl) Benzonitrile (**2f**)

White powder, Yield (96.3%), m.p. = 276–278 °C. IR (KBr, cm^–1^): 3345 (NH), 1594 (C=N), 2224 (C≡N). ^1^H-NMR: δ, ppm (DMSO-*d*_6_); 7.42 (d, 2H, ^3^J = 6.08 Hz, Ar-H), 7.75 (d, 2H, ^3^J = 6.08 Hz, Ar-H), 8.18 (d, 2H, ^3^J = 8.36 Hz, Ar-H), 8.30 (d, 2H, ^3^J = 8.36 Hz, Ar-H), 12.98 (s, 1H, NH, exchange with D_2_O). ^13^C NMR: *δ*, ppm (DMSO-*d*_6_): 115.2 (C≡N), 124.9, 127.8, 129.9, 130.6, 130.8, 133.5, 136.1, 149.5., 167.0 (C=N, imidazole and Ar-C). Anal. Calcd. for C_14_H_9_N_3_ (219.2): C, 76.70; H, 4.14; N, 19.17. Found: C, 76.74; H, 4.16; N, 19.20.

#### 3.1.7. 4-(1H-benzo[d]imidazol-2-yl)-2-methoxyphenol (**2g**)

Beige powder, Yield (52.1%), m.p. = 240–242 °C. IR (KBr, cm^–1^): 3353 (NH), 1590 (C=N). ^1^H-NMR: δ, ppm (DMSO-*d*_6_); 3.73 (s, 3H, OCH_3_), 6.95 (d, 2H, ^3^J = 8.16 Hz, Ar-H), 7.17 (d, 2H, ^3^J = 6.08 Hz, Ar-H), 7.47–7.75 (m, 4H, Ar-H), 12.58 (s, 1H, NH)). ^13^C NMR: δ, ppm (DMSO-*d*_6_): 56.13, 110.7, 113.7, 114.5, 116.1, 118.6, 120.1, 121.7, 146.0, 148.3, 148.9, 152.2 (C=N, imidazole and Ar-C). Anal. Calcd. for C_14_H_12_N_2_O_2_ (224.3): C,74.98; H, 5.39; N, 12.49. Found: C, 74.95; H, 5.40; N, 12.51.

#### 3.1.8. 4-(1H-benzo[d]imidazol-2-yl) Benzoic Acid (**3**)

A mixture of **2f** (10 mmol) and sulfuric acid 60% (30 mL) was stirred at 140 °C for 6 h, then suspended in 150 mL water and the resulting precipitate was filtered off. Recrystallization from ethanol afforded white solid. This compound was prepared in traditional methods [35,36]. Yield (76%), m.p. = 300–302 °C. IR (KBr, cm^–1^): 3373 (br, OH and NH), 1695 (C=O, acid), 1600 (C=N). ^1^H-NMR: δ, ppm (DMSO-*d*_6_): 7.60–7.70 (m, 4H, Ar-H), 8.00 (d, 2H, ^3^J = 8.40 Hz, Ar-H), 8.33 (d, 2H, ^3^J = 8.40 Hz, Ar-H), 10.25 (s, H, OH), 13.19 (s, 1H, NH). Anal. Calcd. for C_14_H_10_N_2_O_2_ (238.24): C, 70.58; H, 4.23; N, 11.76. Found: C, 70.55; H, 4.20; N, 11.74.

#### 3.1.9. Ethyl 4-(1H-benzo[d]imidazol-2-yl) Benzoate (**4**)

To a solution of compound **3** (10 mmol) in absolute ethanol, a few drops of conc. sulfuric acid were added and the mixture was heated under reflux for 4 h. The crude product was filtered off, air dried and recrystallized from ethanol. White powder, Yield (50%), m.p. = 284–286 °C. IR (KBr, cm^–1^): 3334 (NH), 1742 (C=O, ester), 1572 (C=N). ^1^H-NMR: δ, ppm (DMSO-*d*_6_): 1.35 (t, 2H, ^3^J = 7. 20 Hz OCH_2_CH_3_), 4.37 (q, 2H, ^3^J = 7.20 Hz, OCH_2_CH_3_), 7.41 (d, 2H, ^3^J = 7.20 Hz, Ar-H), 7.76 (d, 2H, ^3^J = 7.20 Hz, Ar-H), 8.18 (d, 2H, ^3^J = 8.40 Hz, Ar-H), 8.32 (d, 2H, ^3^J = 8.40 Hz, Ar-H), 13.12 (s, 1H, NH, exchange with D_2_O). ^13^C NMR: δ, ppm (DMSO-*d*_6_): 14.61, 61.65, 115.2, 124.8, 127.8, 130.4, 130.9, 132.4, 133.6, 135.8, 136.3, 149.4, 165.5, 167.0 (C=N, benzimidazole and Ar-C). Anal. Calcd. for C_15_H_14_N_2_O (238.3): C, 75.61; H, 5.92; N, 11.76. Found: C, 75.67; H, 5.91; N, 11.73.

#### 3.1.10. 4-(1H-benzo[d]imidazol-2-yl)benzohydrazide (**5**)

To a solution of ester compound **4** (10 mmol) in ethanol (25 mL), hydrazine hydrate (98%; 2 mL) was added and heated under reflux for 5 h. The reaction mixture was cooled. The crude product was filtered, washed with water and dried. It was crystallized from ethanol. White powder, Yield (86%), m.p. = 270–272 °C. IR (KBr, cm^–1^): 3411, 3303, 3158 (NH_2_/NH), 3014 (arom.-CH), 1594 (C=N); ^1^H-NMR: δ, ppm (DMSO-*d*_6_): 4.35 (s, 2H, NH_2,_), 7.25 (s, 1H, NH, 7.25–8.33 (m, 4H, Ar–H); 12.52 (s, 1H, NH).^13^C NMR: δ, ppm (DMSO-*d*_6_):112.7, 119.6, 122.50, 123.61, 127.5, 130.22, 131.07, 134.77, 135. 59, 140.90, 150.49 (C=N, benzimidazole) and 165.79 (C=O amide). Anal. Calcd. for C_14_H_12_N_4_O (252.27): C, 66.65; H, 4.79; N, 22.21. Found: C, 66.62; H, 4.80; N, 22.23.

### 3.2. Preparation of ZnO

In the current work, a simple sol gel method was used to create uniform nanorods with a hexagonal structure of pure ZnO nanoparticles using zinc acetate Zn(CH_3_CO_2_)_2_ as a metal precursor and sodium hydroxide as a reducing agent. ZnO nanoparticles were synthesized using 50 mL of aqueous 0.1 M NaOH solution [99% (Merck, Darmstadt, Germany] and 500 mL of aqueous precursor 0.05 M zinc acetate solution [ZnAc]. 2H_2_O, Qualikems Reagent (99%) were separately prepared using deionized water after equations 1 and 2. In this experiment, all chemical reagents were attained from commercial sources as guaranteed-grade and used as received devoid of additional treatment. NaOH solution was slowly added to the zinc acetate solution, while vigorous magnetic stirring was conducted at 50 °C to form intensive transparent white gel. The reaction mixture was persistent for 1 h, then allowed to stand at ambient temperature for 24 h. The solution was centrifuged at 3500 rpm for 10 min and supernatant was removed. The gained precipitate was dried in a dryer at 70 °C for 12 h. Consequently, the obtained powder was finally grinded into fine powder.

Zn(CH_3_COO)_2_ ⋅ 2H_2_O + 2NaOH → Zn(OH)_2_ + 2CH_3_COONa + 2H_2_O (1)

Zn(OH)_2_ → ZnO + H_2_O (2)

#### Characterization of the ZnO

The prepared ZnO nanoparticles were characterized by XRD, SEM/EDX and TEM. The crystalline phase of ZnO was identified using X-ray diffraction analysis via a [Bruker D8 advance diffractometer, Germany] with Cu-K_α_ radiation (λ = 0.15418 nm). The aim of using the XRD was to evaluate the phase purity and growth of crystalline phases, and consider the nanosize of the prepared ZnO. The X-ray diffractometer functioned at 40 kV and 40 mA in the range of 2θ (10–70°). The crystalline phase was demonstrated by comparing the diffraction patterns of the fabricated composites with Joint Committee on Powder Diffraction Standards (JCPDS) standards.

The microcrystalline and morphology configuration of the ZnO was proved via the scanning electron microscope equipped with energy dispersive X-ray microanalysis (SEM/EDX, model FEJ Quanta 250 Fei, Eindhoven, The Netherlands) operating at voltage 15 kV. The samples’ surface was layered by gold by means of a [S150A sputter coater, Edwards, England] under 0.1 Torr, vacuum 1.2 kV voltage and 50 mA current. The purpose of this coating was to improve the scanning of samples.

As well, the nanoparticle nature and crystallinity were participated observed by high resolution transmission electron microscope (HR-TEM, Joel model JEM-2100, operating voltage 200 kV, Sendai, Japan). Aqueous dispersion of the particles was drop-casted onto a copper grid coated with carbon before being air dried at room temperature to be microscopically scanned.

The zeta potential of the prepared sample was measured using (Zetasizer, Nano-ZS, Malvern Instruments Ltd., Malvern, UK). Prior to zeta assessment, about 0.05 g of the sample was dispersed and sonicated in 5 mL distilled water for 30–60 min in a bath ultrasonicator. The constancy of the suspensions will be examined by measuring the zeta potential. The refractive indexes for ZnO and dispersant (water) were agreed to be 2.00 and 1.33, correspondingly.

### 3.3. DPPH Radical Scavenging Activity

A freshly prepared (0.004% *w*/*v*) methanol solution of 2,2-diphenyl-1-picrylhydrazyl (DPPH) radical was prepared and stored at 10 °C in the dark. A methanol solution of the test compound was prepared. A 40 μL aliquot of the methanol solution was added to 3 mL of DPPH solution. Absorbance measurements were recorded immediately with a UV-visible spectrophotometer (Milton Roy, Spectronic 1201). The decrease in absorbance at 515 nm was continuously determined, with data being recorded at 1 min intervals until the absorbance stabilized (16 min). The absorbance of the DPPH radical without antioxidant (control) and reference compound ascorbic acid were also measured. All the determinations were performed in three replicates and averaged. The percentage inhibition (PI) of the DPPH radical was calculated according to the formula:

PI = [{(AC − AT)/AC} × 100](3)

where AC = Absorbance of the control at t = 0 min and AT = absorbance of the sample+DPPH at t = 16 min [32].

The 50% inhibitory concentration (IC_50_), the concentration required to 50% DPPH radical scavenging activity was estimated from graphic plots of the dose response curve using Graphpad Prism software (San Diego, CA, USA; https://www.graphpad.com/features accessed on 30 May 2023).

#### Antioxidant Evaluation

The antioxidant activity was performed using the DPPH radical scavenging method where ascorbic acid was used as a positive control for comparison [32]. The results of the antioxidant activity of the compounds **2a**, **2c**, **2f**, **3**, **4** and **5** are shown in Table 2. The activity was assessed by measuring its electron-donating ability to DPPH, which is indicated by changes in absorbance of the solution of different concentrations at 515 nm. The DPPH radical scavenging activity of the compounds increased with an increase in concentration; the result of the radical scavenging was expressed in terms of half-inhibition concentration (IC_50_), which denotes the concentration required to scavenge 50% of DPPH radicals.

### 3.4. Insilico Studies

#### 3.4.1. Molecular Docking (Antioxidant)

The computational methods for the most bioactive compounds that would be docked utilizing Molecular Operating Environment software (2015) were developed using Chemdraw 12.0. The data were evaluated using the London DG force and force field energy. MMFF 94 (Merck molecular force field 94) was used for all minimizations up until a root mean square deviation (RMSD) gradient of 0.1 kcalmol^−1^ A^−1^ was reached [37,38], and automatic estimation of partial expenses. The MOE program’s dock function (S, kcal/mol) was utilized to evaluate the ligand’s ability to bind.

The protein data bank provided the X-ray crystal structure of the enzyme in PDB format (PDB ID: 2X08, resolution: 2.01) (https://www.rcsb.org/structure/2X08 (accessed on 30 May 2023)). In order to prepare the enzyme for docking studies, water was removed, all hydrogen bonds were added, the potential was fixed, and fake atoms were created from the resulting alpha spheres [39]; and how the ligand interacts with the active site’s amino acids examined. The best Docking Score is produced by the active ligand’s biggest negative value [40,41,42].

#### 3.4.2. Assessment of Pharmacokinetic Properties

The oral bioavailability characteristics of the compounds were investigated using the SwissADME website, and key characteristics relating to the drug-likeness of the chosen compounds were assessed using the online tool Molinspiration (http://molinspiration.com/ (accessed on 30 May 2023)) [43,44].

#### 3.4.3. Molinspiration

##### Bioavailability Radar

It is a tool for quickly evaluating a molecule’s drug-likeness. Six physicochemical characteristics were taken into account: lipophilicity, size, polarity, solubility, flexibility, and saturation. A physicochemical range was established on each axis using descriptors, as previously mentioned [45].

##### Physicochemical Properties

These include straightforward molecular and physicochemical descriptors that indicate the complexity of the molecule, such as molecular weight (M.W.), the number of particular atom types, fraction Csp3 (carbon bond saturation as defined by fraction sp3) and molecular refractivity (M.R.). Additionally, the polar surface area (PSA), which was calculated using the topological polar surface area (TPSA), was utilized to swiftly estimate several ADME features, particularly those related to passing through biological barriers, including absorption and brain entrance [46].

##### Lipophilicity

The consensus log Po/w, which is the mean of the values predicted by the five free predictors iLOGP, XLOGP3, WLOGP, MLOGP and SILICO- IT, is then generated by Molinspiration [47].

##### Water Solubility

Water Solubility is predicted by Molinspiration. A bioavailability score was developed [48]. The results are the decimal Log p of the molar solubility in water and the Log S values. Additionally, the qualitative solubility classes and water solubility in mg/mL and mol/L were reported.

##### Pharmacokinetics

Molinspiration employs specific models to assess the test compound’s ADME characteristics. Christopher A. Lipinski developed the “Lipinski Rule of Five” in 1997 as a guideline for evaluating drug-likeness and deciding whether an inhibitor with particular biological and pharmacological characteristics would be an orally active medication in the human body. According to the rule, a molecule can be orally absorbed/active if two (2) or more of the following thresholds are met: molecular weight (Mw) of the molecule (500), octanol/water partition coefficient (ilog P)_5, number of hydrogen bond acceptors (nHBA)_10, number of hydrogen bond donors (nHBD)_5, and topological polar surface area (TPSA) 40 2. The first model forecasts blood-brain barrier (BBB) penetration and passive gastric absorption [49]. The second approach predicts whether or not the permeability glycoprotein (P-GP), which is necessary to assess active efflux through membranes, such as from the gastrointestinal wall to the lumen or brain, is a substrate or non-substrate [50]. The third model predicts the interaction of substances with the key isoenzymes of cytochrome P450 (CYP) (CYP1A2, CYP2C19, CYP2C9, CYP2D6, CYP3A4), which are crucial for the metabolic biotransformation process that leads to drug clearance. Additionally, these isoenzymes’ inactivation contributes to medication interactions [46] causing poisonous or other negative effects. The skin permeability coefficient (Kp), which is linearly associated with molecule size and lipophilicity, is predicted by the fourth model [50]. Skin permeability decreases as log Kp (cm/s) becomes increasingly negative.

### 3.5. Computational Details

The geometric parameters and energies were computed by density functional theory at the B3LYP/CEP-31G level of theory, using the GAUSSIAN 98 W package of the programs [51], on geometries that were optimized at CEP-31G basis set. The high basis set was chosen to detect the energies at a highly accurate level. The atomic charges were computed using the natural atomic orbital populations. The B3LYP is the keyword for the hybrid functional [52], which is a linear combination of the gradient functionals proposed by Becke [53] and Lee, Yang and Parr [54], together with the Hartree-Fock local exchange function [55].

#### Structural Parameters and Models

##### Compounds of **2a**–**2g** and (**3**–**5**)

The geometrical structures of all studied compounds are slightly sterically-hindered; the atoms of these compounds are distributed in the one plane as shown in Figures S1–S10 (See Supplementary Materials). The planarity of these compounds plays an important role in the rising of the degree of stability, and also effects the degree of biological effect of these compounds. The complete planarity of these compounds also causes lowering of the values of the dipole moments of these compounds. The dihedral angles are varied from 0.0° to 180.0° for all studied compounds; these values confirm that the two aromatic systems are lying in the same plane. The bond angles are varied between 105° to 127°; these values reflect the type of sp2 hybridization spreading over most atoms of the studied compounds; all bond angles and dihedral angles are listed in Table S1 for the compounds **2a**–**2g** and Table S2 for compounds **3**–**5**.The planarity of the molecules responsible for its activities and also in the biological activity, the substituent aromatic system and benzimidazole can be rotated around the carbon-carbon bonds, C8–C10, but it does not occurred and molecules favor presence as planer molecules without any rotation to the one occupied plane. The value of energy of these compounds is varied between −7275.761 kcal/mol for the compound (**2b**), which is less stable and −11,082.178 kcal/mol for the compound (**2c**), which is considered more stable than other studied compounds as listed in the Table S3, while the value of energy for the compounds **3**, **4** and **5** are −9559.620, −11,197.394 and −10,255.360 kcal/mol, respectively. Also, the values of the dipole moment are varied between 4.442 D for compound (**2g**) and 5.905 D for compound (**2a**); also, the other compounds **3**–**5** possess lower dipole moment values than the others. The dipole moment values of these compounds as listed in Table S2 are varied from 1.680 to 3.193 D. The bond length between the two aromatic systems C8-C10 in all studied compounds are varied between 1.339 to 1.355 [56] Å, which are the shortest C-C bonds among all C-C bonds. The bond lengths, C8-N9 and N7-C8 of the benzimidazole ring are varied between 1.351 and 1.354 [57] Å for C8-N9; and between 1.339 and 1.343 [56] Å, there is a single bond character between N7 and C6 atoms; and also between N9 and C5 atoms [58,59] the bond lengths between nitrogen atom N9 and the neighbor carbon atoms C8 of the benzimidazole ring have double bond characters [60]; while the bond lengths between nitrogen atom N7 and the neighbor carbon atoms C8 of the benzimidazole ring have single bond characters. The bond lengths between atoms are listed in the Tables S1 and S2. These values are compared with the crystal structure of the molecule, which has a similar structure [61]. Detailed analysis of corresponding bond lengths in various hetero-cyclic compounds was given elsewhere [57,61].

##### Molecular Orbitals and Frontier

Molecular orbitals also play an important role in the electric properties, as well as in UV-Vis [62]. An electronic system with smaller values of the HOMO-LUMO gap should be more reactive than one having a greater energy gap [63]. The energy gap, ΔE of the studied compounds, varied between 0.039 for compound (**2a**) which is more reactive; and 0.149 eV for compound (**2b**), which is less reactive. So electron movement between these orbitals could easily occur by decreasing the value of the energy gap, so that there is a peak around 250 nm in the UV-Vis spectra for all studied compounds. On the other hand, the adjacent orbitals are often closely spaced on the frontier region.

**Figure 9:**
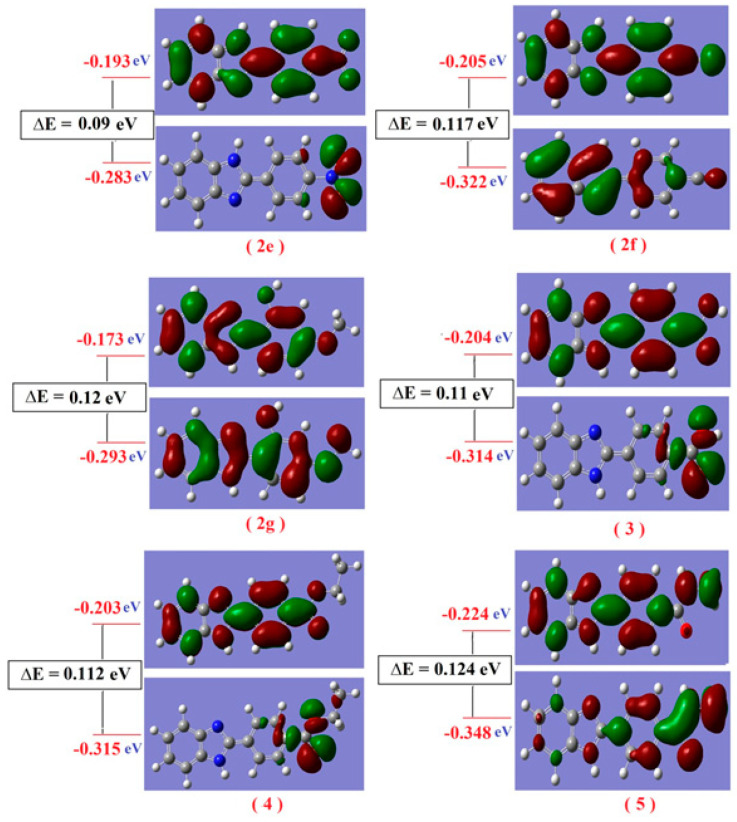
Molecular orbital surfaces and energy levels of compounds (**2e**–**2g**) and (**3**–**5**) by using DFT calculations.

The nodal properties of molecular orbitals of the studied compounds in Figure S11 are illustrative and suggest orbital delocalization, strong orbital overlap, and a low number of nodal planes. The energy difference between HOMO and LUMO (energy gap, ∆E) for all studied compounds varied according to the type of substitutions as shown in Table S3. Hard molecules have a higher HOMO-LUMO gap, and soft molecules have a smaller HOMO-LUMO gap [64]; the degree of softness is important in the detection of the biological activity of any compound, whereas the greater softness value means the greater biological activity value. The values of η and ∆E (HOMO-LUMO) are given in Table S3. It is obvious that the compound (**2a**) is softer than all studied compounds; the value η varied from 0.019 for compound (**2a**) to 0.075 for compound (**2b**). Also, the electronic transition within the soft compounds is easy as indicated from the ∆E. There are some quantum chemical parameters depending upon the energy values of HOMO and LUMO were calculated as global softness (S), electro negativity (χ), absolute softness (σ), chemical potential (Pi), global electrophilicity (ω) and additional electronic charge (∆N_max_) of all studied compounds. From these values, the compound (**2a**) is absolute soft according to the (σ = 25.641 eV), while the compound (**2b**) is treated as hard compounds (σ = 13.333 eV). As seen in Figure S11, all compounds have the same nucleus aromatic systems and different substitution. These compounds are divided into different parts according to composition as given in Table S4; there are three parts, the benzene ring, imidazole ring and substitution group. The electron density of the HOMO state of all studied compounds is delocalized over all atoms with different portions; except in the case of (**2e**, **3** and **4**), where there is a localization of the electron density mainly on the substitution group with 100% without any percent on any atoms of the benzimidazole system with 0.0%. The electron density of the LUMO is also delocalized over all atoms of the compounds with different portions, as in Figure 9.

##### Charge Distribution Analysis

The charge distribution analysis on the optimized geometry configuration of all studied compounds was made on the basis of natural population analysis (NPA). Selected data are reported in Table S5. The charge distribution accumulated on the benzimidazole ring was affected by the changing substituent group; in the case of compound (**2a**), there is a lower negative charge on the benzimidazole ring than on others; and also, there is a higher negative charge on the substituent group. These values indicate there is a net negative pole and a net positive pole on the molecule; as a result, the molecule is more dipole, μ = 5.905 D. The charge density accumulated on the benzimidazole varied from −0.205 for compound (**2a**) to −0.3075 for compound (**2b**); the charge accumulated on the substituent groups varied from −0.0399 for compound (**2b**) to −0.5723 for compound **(5**). The most negative charge is localized on the nitrogen atoms of the benzimidazole ring system and localized on the nitrogen, oxygen and sulfur atoms of the substituent, while all hydrogen atoms in all compounds carry a positive charge. These results mean that electronic transitions π-π* and n-π* can be carried out from a higher electron density region toward the lower electron density regions involved in these compounds according to its composition. This conclusion is further confirmed by comparing the values of the calculated charge density on the donating nitrogen atoms of the benzimidazole ring and also the oxygen, nitrogen and sulfur atoms of the substituent in different compounds. The distribution of atomic charges is also important in the determination of the direction of the dipole moment vector in the compounds, which depends on the centers of negative and positive charges.

##### Excited State

The TD-DFT at the B3LYP levelusing the G03W program proved to give an accurate description of the UV-vis. Spectra [65,66]. Time-dependent density functional response theory (TD-DFT) has been recently reformulated [67] to compute discrete transition energies and oscillator strengths, and has been applied to a number of different atoms and molecules. Bauernschmitt and Ahlrichs [68] included hybrid functionals proposed in the calculation of the excitation energies. The electronic transition could be described as mixed π-π* and n→π* transitions. The energies of HOMO and LUMO states for all studied compounds are listed in Tables S6–S8. The HOMO can perform as an electron donor and the LUMO as the electron acceptor in a reaction profile.

Six excited states are involved in the case of compound (**2a**); these excited states corresponding with four absorption bands were obtained experimentally as given in Table S6. The first experimental band was obtained at λmax = 333.5 nm; this band is composed of two exited states results from a combination of several transitions, H-2 → L (17%), H-2 → L + 1 (9%), H-2 →L + 2 (23%), H-1 → L + 2 (21%), H-1 → L + 3 (15%), H → L + 1 (13%), H → L + 2 (19%), this excited state assigned to π-π* at 327.6 nm and H-4 →L (15%), H-2→L+1 (49%), H-1 → L + 3 (17%), H → L + 2 (9%) at 338.96 nm and assign to π-π*. The second observed band at 342 nm is composed of the combination of two excited states. The first excited state results from interaction between electronic configurations at 340.83 nm, which represent n→π* transition. This transition results from H-4 → L + 1 (14%), H-4 → L + 2 (11%), H-2 → L (9%), H-1 → L (12%), H-1 → L + 1 (23%), H-1 → L + 3 (25%), H → L + 1 (24%), H → L + 3 (17%) and the second excited state at 348.28 nm and results from H-4 → L + 1 (13%), H-4 → L + 3 (7%), H-2 → L (14%), H-2 → L + 2 (21%), H-1 → L (16%), H-1 → L + 2 (13%), H → L + 1 (26%), H → L + 3 (8%) which assign to π-π*. The third observed absorbed band at 367 nm was composed of only one excited state H-3 → L (14%), H-3 → L + 4 (12%) at 370.77 nm and assign to π-π* transition. The fourth observed band at 373.5 nm was equivalent to one excited state, which results from H-3 → L + 4 (12%) at 379.35 nm and assigned to π-π* transition. All electronic transition configurations of the compounds (**2a**–**2d**) are given in Table S6, and the absorption spectra of the compounds (**2a**–**2d**) are shown in Figure S12.

In Table S7, in the case of compound (**2e**), seven excited states were obtained theoretically. These excited states correspond with three observed absorption bands as shown in Figure S13. The first excited state results from a combination of several transitions, H-8 → L + 2 (16%), H-5 → L + 2 (13%), H-3 → L (23%), H-3 → L + 3 (27%), H-3 → L + 5 (28%), H-1 → L + 2 (16%), H → L + 2 (13%); this excited state assigned to π-π* at 328.04 nm. The second excited state results from interaction between electronic configurations, which represent n→π* transition; this transition results from H-1 → L (38%), H-1 → L + 1 (23%), H-1 → L + 3 (21%), H → L (16%), H → L + 3 (13%), H → L + 4 (16%) and can be observed around 333.06 nm. The third excited state is assigned to π→π* transition, represents a transition appearing at 336.61 nm, H-2 → L (24%), H-2 → L + 1 (21%). These three excited states are combined together to express on the observed absorption band at 334 nm. The fourth excited state at 358.01 nm results as H-7 → L (23%), H-7 → L + 1 (18%), H-7 → L + 2 (14%) and assigned to n-π*. The fifth exited state results from combination transitions, H-5 → L (21%), H-1 → L (23%), H → L (16%), H → L+1 (79%). This excited state assigned to π→π* at 361.48 nm. These excited states are corresponding with the observed absorption band at 359 nm. The sixth excited state results from interaction between electronic configurations, which represent n→π* transition. This transition results from H-4 → L (21%), H-4 → L + 1 (23%), H-4 → L + 3 (16%) and can be observed around 371.69nm. The seventh excited state is assigned to n→π* transition, and represents a transition appearing at 375.75 nm, H-6 → L (21%), H-6 → L + 1 (23%), H-6 → L + 3 (16%). These excited states correspond with the observed absorption band at 374.5 nm. Also, all electronic transition configurations of the compounds (**2e**–**2g**) are given in the Table S7.

In the case of compound (**3**), there are five observed absorption bands as shown in Figure S13. The first band at 334 nm is combined of one excited state, which results from H-3 → L (16%), H-2 → L (13%), H-2 → L + 2 (23%), H-1 → L + 3 (27%), H-1 → L + 4 (28%), H → L + 1 (16%), H → L + 2 (13%), H → L + 3 (13%), which assigned to π-π* at 331.23 nm. The second observed absorption band at 346 nm was composed of two excited states, one assigned to n→π* transition at 340.21 nm and the other assigned to π→π* transition at 343.26 nm. These transitions result from H-3 → L (38%), H-3 → L+1 (23%), H-3 → L + 2 (21%), H-2 → L (16%), H-2 → L + 2 (13%), H → L + 2 (16%) and H-1 → L (24%), H-1 → L + 1 (21%), H-1 → L + 2 (24%), H → L + 3 (21%). The third observed absorption band at 353.5 nm results from H-5 → L (23%), H-5 → L + 1 (18%), H-5 → L + 4 (14%), H-5 → L + 5 (14%) at 351.69 nm and assigned to n→π* transition. The fourth observed absorption band at 369 nm corresponds with the excited state at 370.4 nm, resulting in H-1 → L (21%), H → L (23%), H→L+1 (79%). The fifth absorption band at 384 nm is assigned to n→π* transition and results from H-4 → L (21%), H-4 → L + 1 (23%), H-4 → L + 3 (16%), H-4 → L + 4 (23%), H-4 → L + 5 (16%) and can be observed around 388.2 nm. All electronic transition configurations of the compounds (**3**–**5**) are given in the Table S8.

## 4. Conclusions

The authors herein endeavored to design an efficient and environmentally friendly protocol to synthesize benzimidazoles derivatives **2a**–**g** and **3**–**4**. The synthesized compounds were produced in excellent yield using ZnO-NPs. In addition, results indicated that compound **2a** has higher in vitro antioxidant activity than those of standard ascorbic acid. The compound **2a** is absolute soft according to this study, and the results revealed that the newly formed compounds exhibited antioxidant activities on HRBC hemolytic and membrane stabilization and DPPH scavenging percent, respectively. All synthesized compound structures were elucidated via the most different elemental and spectral analytical methods. The superiorities of this procedure are environmental, high yield of product and low amounts of catalyst, and short reaction time.

## Figures and Tables

**Table 1:** Optmization of different nanoparticles and their effects on yield of 2-. (1*H*-benzo[*d*]imidazol-2-yl) phenol (**2a**).

Entry	Solvent	Catalysis (mol)	Temp (°C)	Time (h)	Yield (%)	Ref.
1	EtOH	TiO_2_(nano) (0.02%)	70 °C	2	86%	This work
2	EtOH	ZnO(nano) (0.02%)	70 °C	2	92%	This work
3	Toluene	*p*-TsOH	80 °C	2	81%	[19]
4	EtOH	PSA	r.t	30 min	85%	[20]

**Table 2:** Antioxidant activity of 2-substituted benzimidazoles **2a**, **2c**, **2f** and (**3**–**5**).

% Inhibition of DPPH Free Radical at Different Concentrations											
Compds	2 (µg/mL)	3.9 (µg/mL)	7.8 (µg/mL)	15.6 (µg/mL)	31.25 (µg/mL)	62.5 (µg/mL)	125 (µg/mL)	250 (µg/mL)	500 (µg/mL)	1000 (µg/mL)	IC_50_
**2a**	27.41	35.50	51.89	63.76	81.24	88.75	91.86	93.21	93.88	95.63	**7.35**
**2c**	4.8	7.39	10.34	16.58	26.23	40.61	44.45	84.73	90.13	92.82	85.21
**2f**	1.23	2.85	5.91	8.23	15.96	27.18	36.40	50.38	72.54	89.75	246.60
**3**	2.86	5.37	8.94	16.45	25.60	34.87	55.49	73.62	89.05	92.18	108.35
**4**	1.74	3.28	8.72	14.59	21.53	31.76	48.45	57.12	69.75	84.06	147.34
**5**	4.31	6.89	14.63	20.82	36.29	44.15	69.23	82.75	88.24	91.89	77.07
**Ascorbic acid**	22.18	1.04	43.01	65.05	79.35	86.42	90.89	94.25	96.34	98.65	10.21

**Table 3:** The binding scores, RMSD values, distance and receptor interactions of the three most promising compounds (**2a** and **5**) compared to the docked reference ascorbic acid for antioxidant activity.

Comp.	Score (Kcal/mol)	RMSD	Interacting Residues		
			Receptor Interactions	Distance (Å)	E (Kcal/mol)
**Ascorbic acid**	**−7.0409**	1.5912	Asp37/H-donor Gly41/H-donor Val45/H-acceptor Arg184/H-acceptor	2.58 2.68 2.81 2.74	−4.90 −1.40 −2.90 −4.40
**2a**	−7.3817	1.4893	Ser81/pi-H	3.91	−0.90
**5**	−7.1462	2.0053	His181/H-donor	3. 39	−1.20

**Table 4:** **a**—Physicochemical properties of the synthesized compounds. **b**—Physicochemical Molinspiration bioactivity score.

(**a**)									
**Compound**	**miLogP**	**TPSA**	**n-Atoms**	**M.W.**	**nON**	**nOHNH**	**n-Violations**	**n-rotb**	**Volume**
**2a**	3.28	48.91	16	210.24	3	2	0	1	188.29
**5**	1.93	83.80	19	252.28	5	4	0	2	222.95
(**b**)									
**Compound**	**GPCR Ligand**	**Ion Channel Modulator**		**Kinase Inhibitor**		**Nuclear Receptor Ligand**		**Protease Inhibitor**	**Enzyme Inhibitor**
**2a**	−0.10	0.10		0.11		−0.25		−0.47	0.16
**5**	−0.16	−0.48		0.15		−0.64		−0.24	0.07

## Data Availability

All data generated or analyzed during this work are available from the correspondence author on request.

## Supplementary Materials

The following supporting information can be downloaded at: https://www.mdpi.com/article/10.3390/ph16070969/s1, Figure S1: IR Spectrum of compound (**2a**). Figure S2: 1H-NMR Spectrum compound of (**2a**). Figure S3: 13C-NMR Spectrum of compound (**2a**). Figure S4: IR Spectrum of compound (**2c**). Figure S5: 1H-NMR Spectrum of compound (**2c**). Figure S6: 13C-NMR Spectrum of compound (**2c**). Figure S7: IR Spectrum of compound (**2d**). Figure S8: 1H-NMR Spectrum of compound (**2d**). Figure S9: 13C-NMR Spectrum of compound (**2d**). Figure S10: IR Spectrum of compound (**2e**). Figure S11: 1H-NMR Spectrum of compound (**2e**). Figure S12: 13C-NMR Spectrum of compound (**2e**). Figure S13: 1H-NMR Spectrum of compound (**2f**) in D2O. Figure S15: 13C-NMR Spectrum of compound (**2f**). Figure S17: 1H-NMR Spectrum of compound (**3**). Figure S19: 1H-NMR Spectrum of compound (**4**) in D2O. Figure S20: 13C-NMR Spectrum of compound (**4**). Figure S23: 1H-NMR Spectrum of compound (**5**). Figure S24: 13C-NMR Spectrum of compound (**5**).
