# Supplementary material for: Eco-Friendly Synthesis of 1H-benzo[d]imidazole Derivatives by ZnO NPs Characterization, DFT Studies, Antioxidant and Insilico Studies
Source: Pharmaceuticals (Basel). 2023 Jul 6;16(7):969. doi: 10.3390/ph16070969 (PMC10385378; doi:10.3390/ph16070969)
Supplement: Supplementary file 1 [file pharmaceuticals-16-00969-s001.zip › pharmaceuticals-2459091-supplementary.pdf]

SPECTRAL ANALYSIS

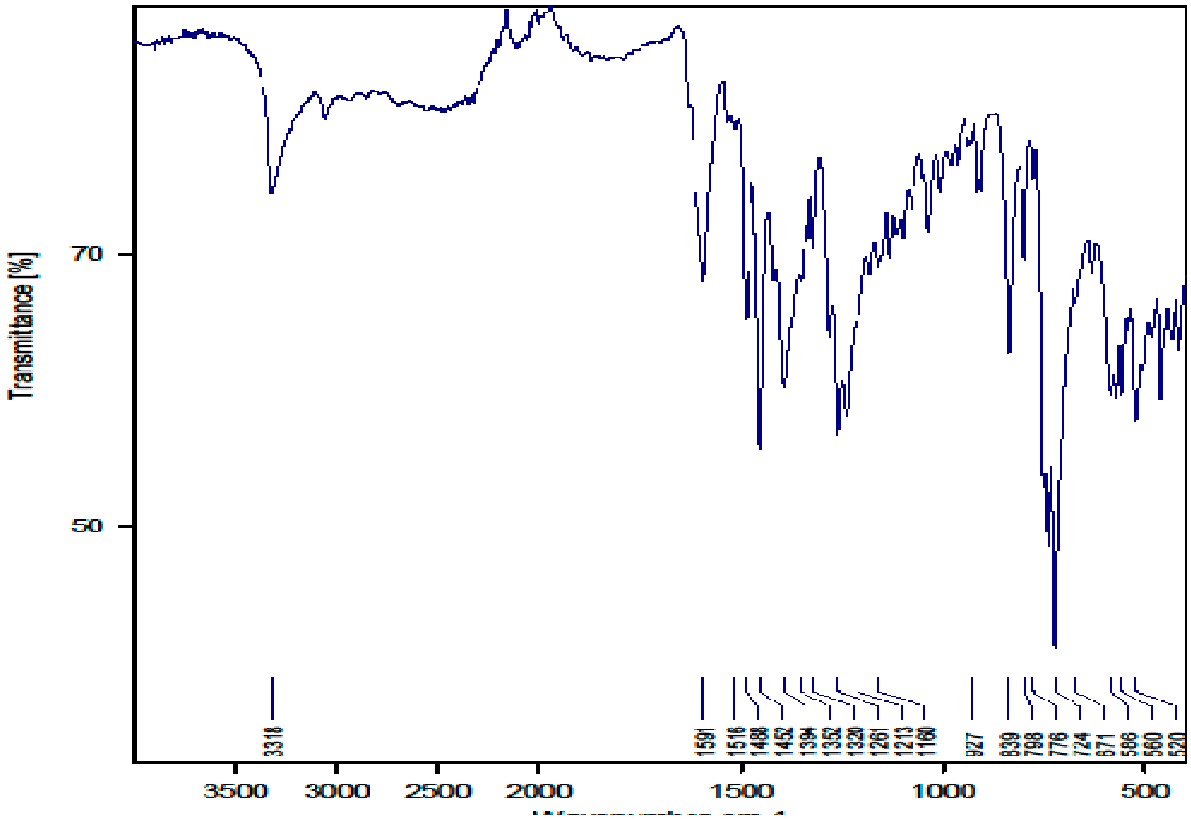

Figure S1: IR Spectrum of compound (2a)

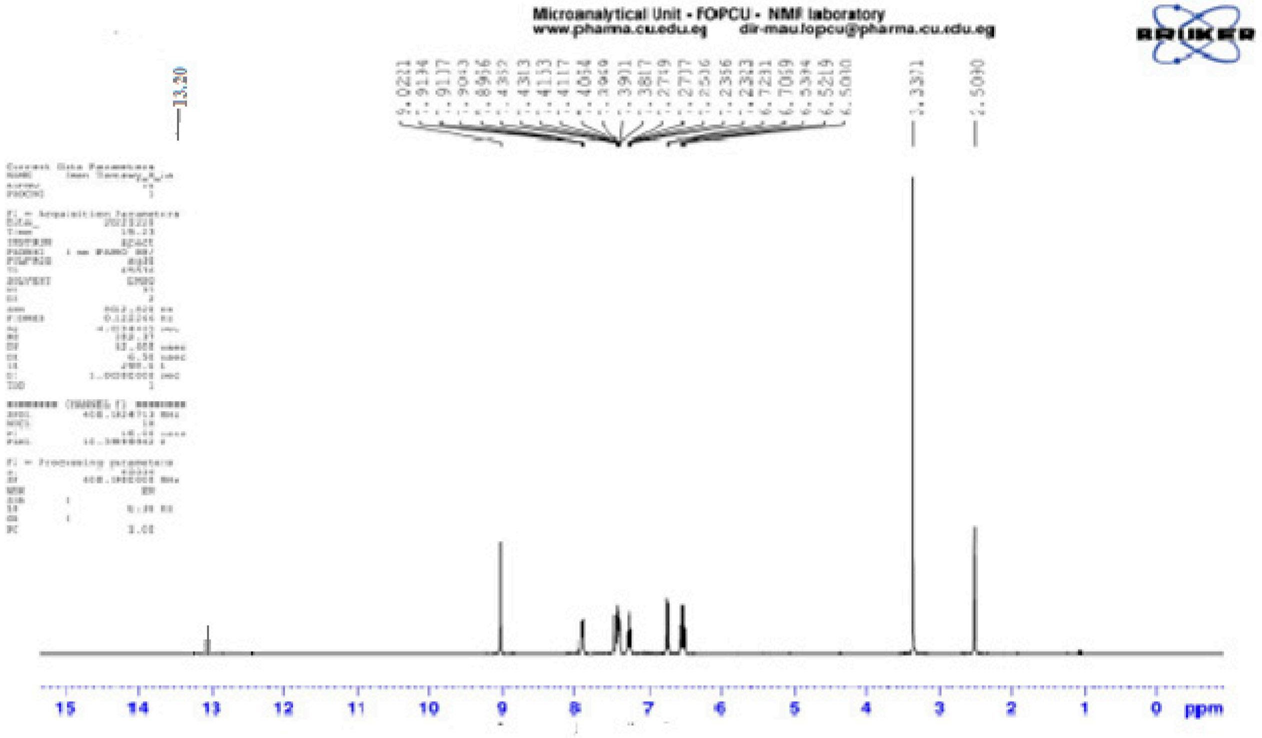

Figure S2: ^{1}H-NMR Spectrum compound of (2a)

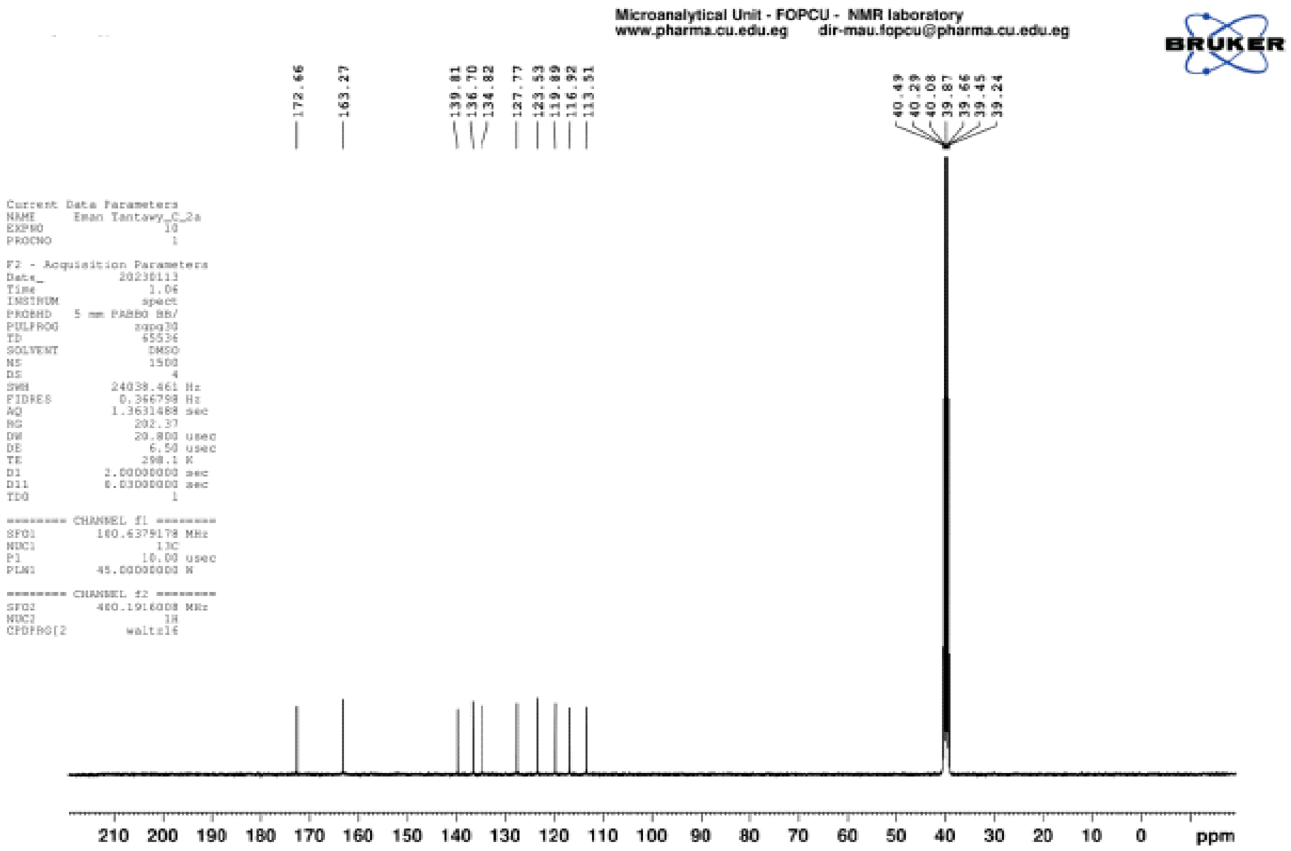

Figure S3: ^{13}C-NMR Spectrum of compound (2a)

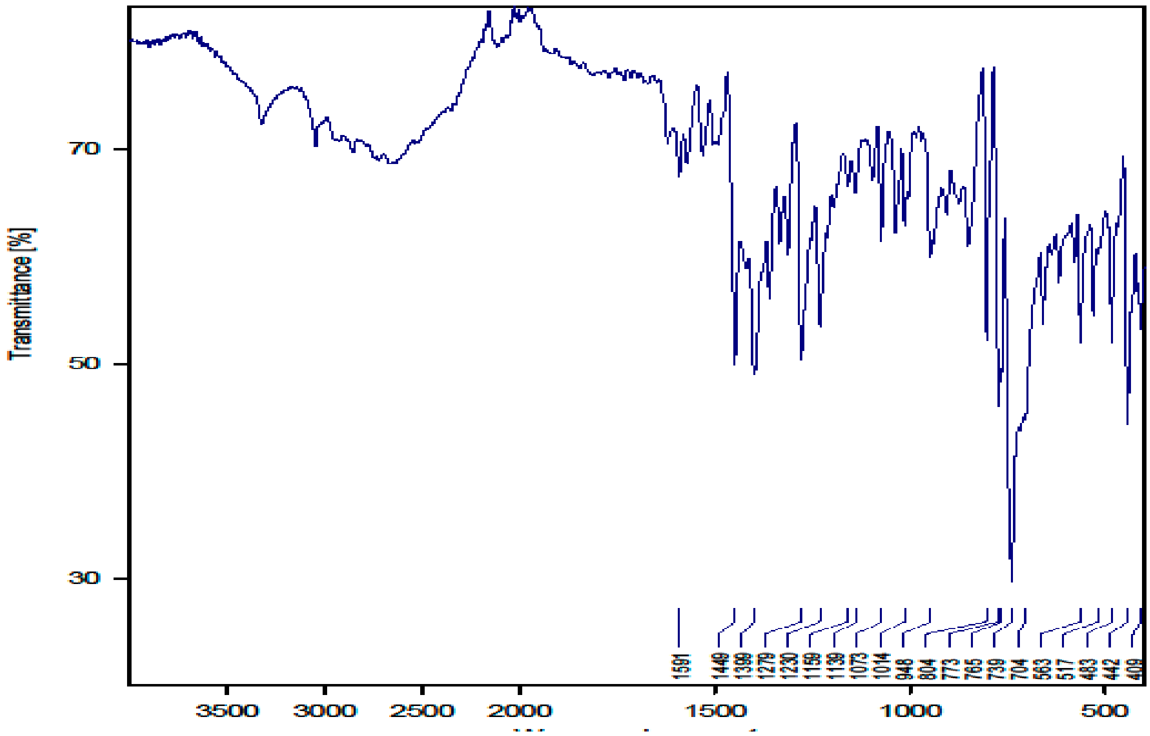

Figure S4: IR Spectrum of compound (2c)

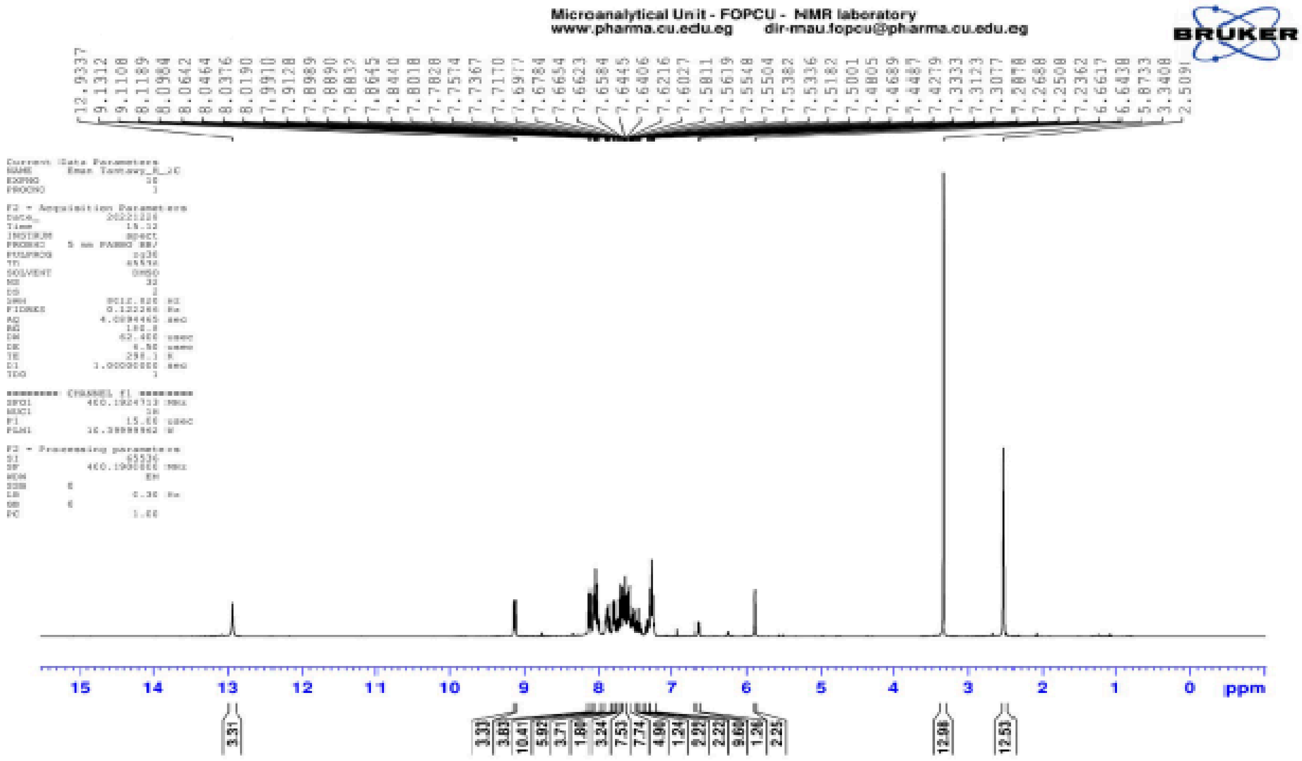

Figure S5: ^{1}H-NMR Spectrum of compound (2c)

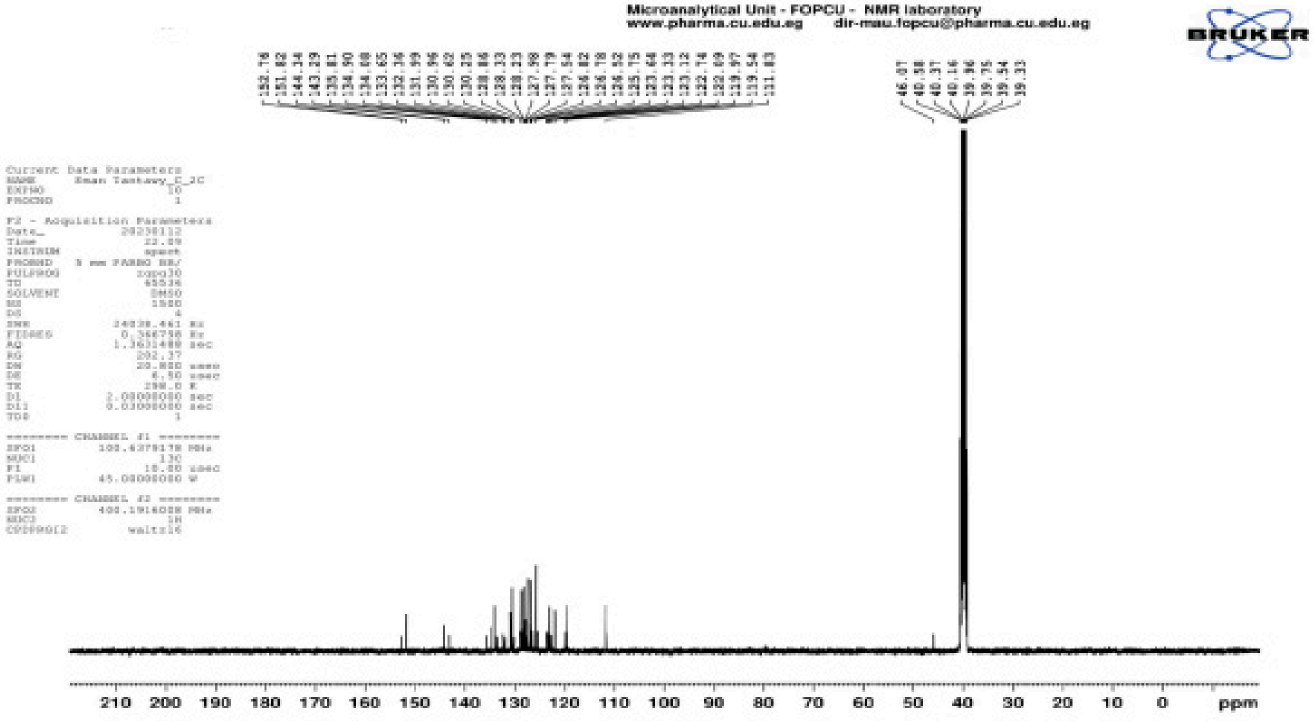

Figure S6: ^{13}C-NMR Spectrum of compound (2c)

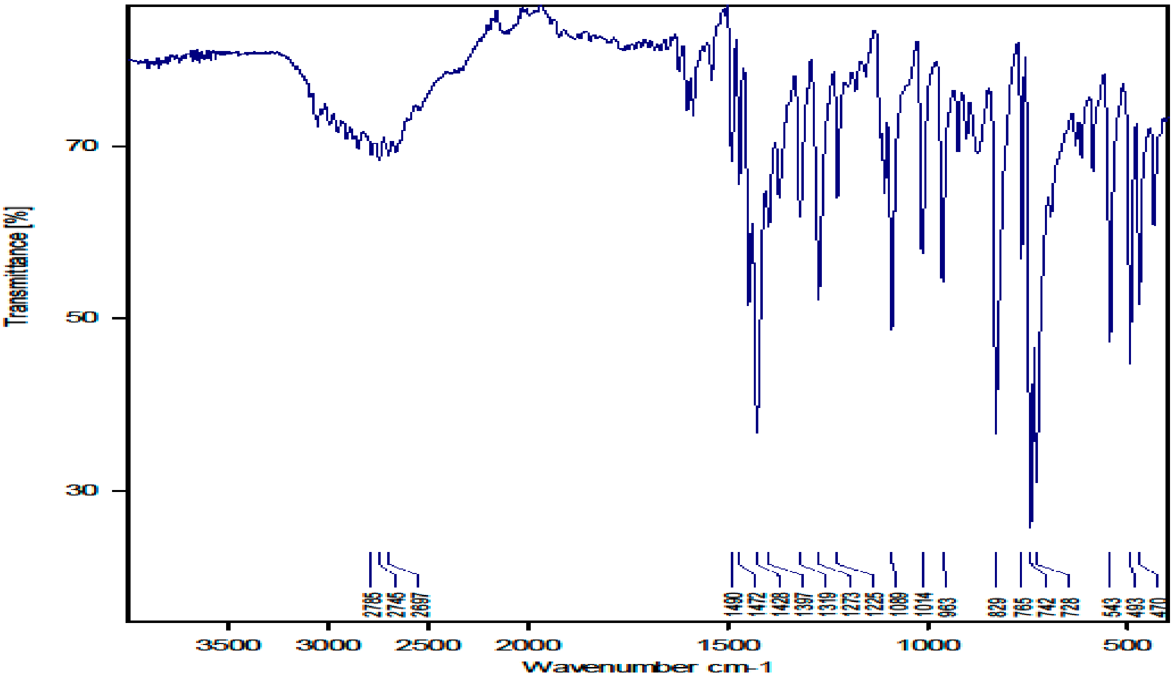

Figure S7: IR Spectrum of compound (2d)

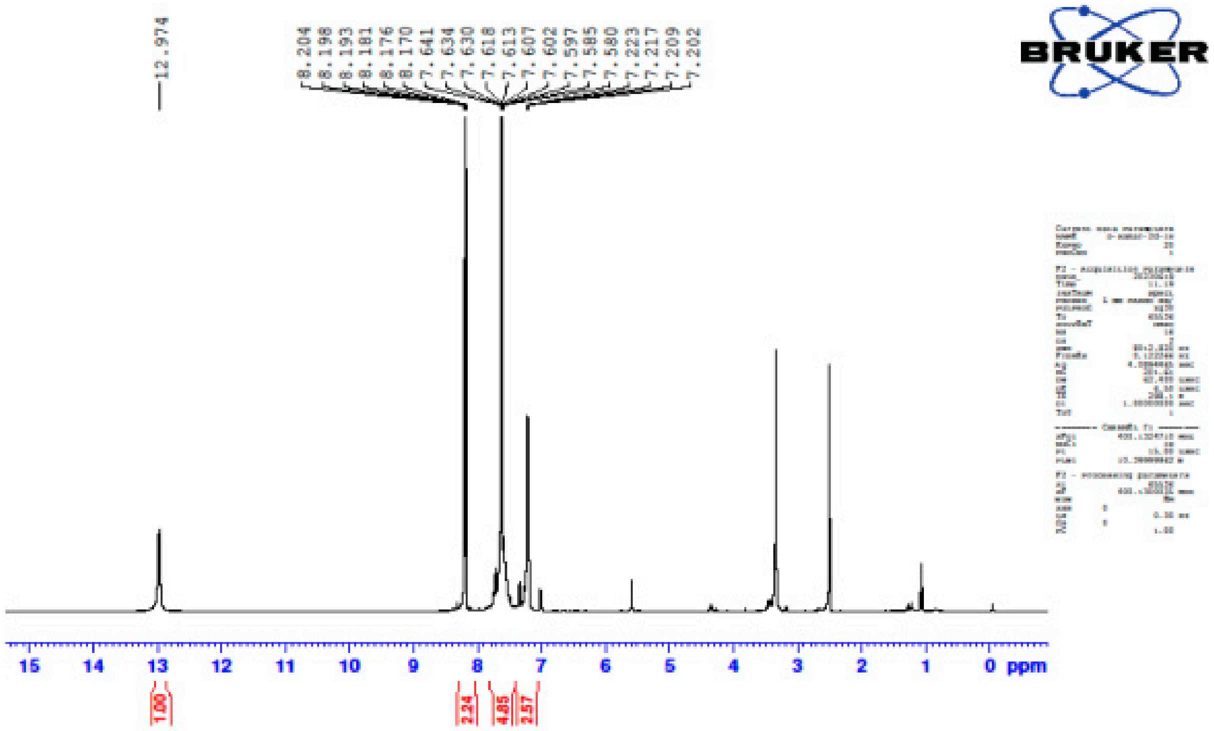

Figure S8: ^{1}H-NMR Spectrum of compound (2d)

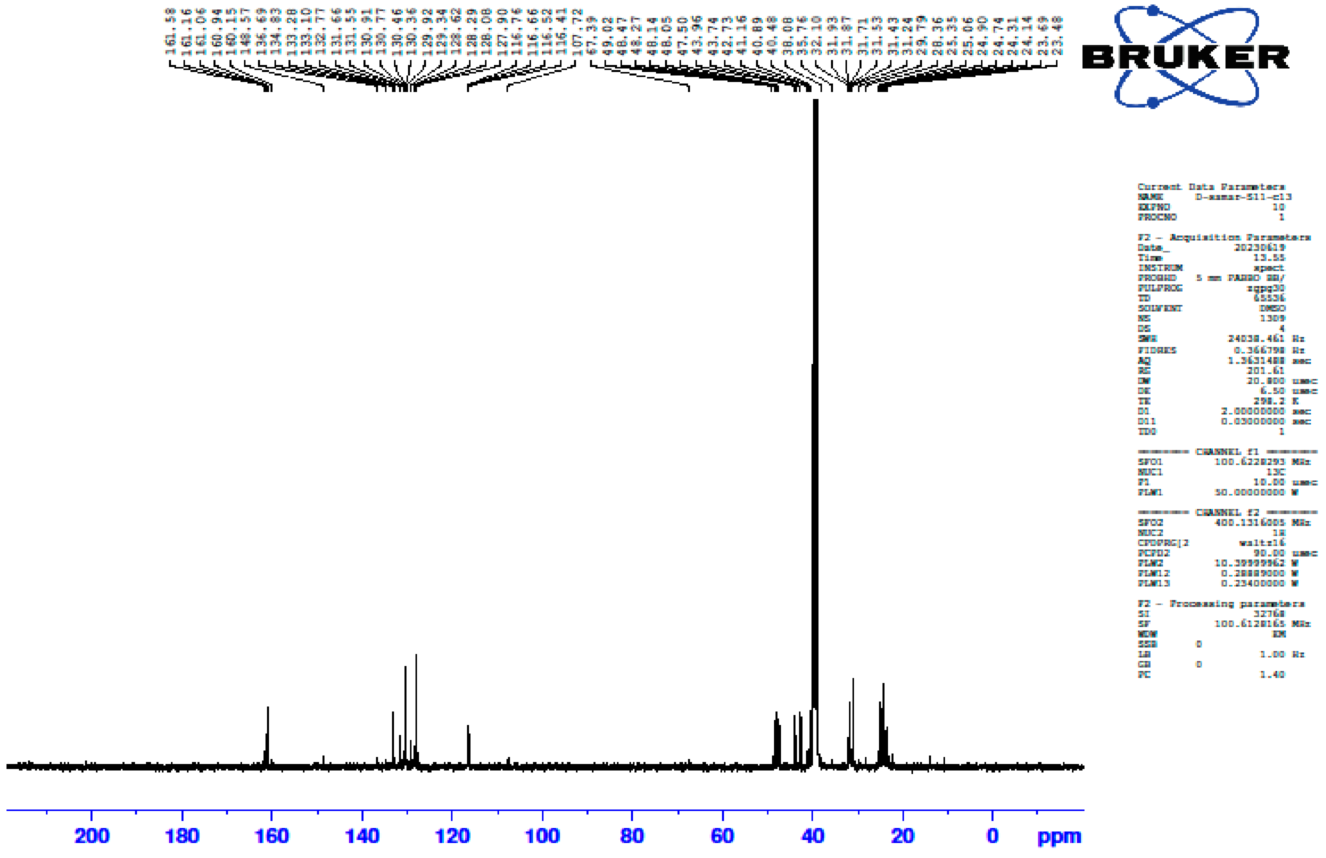

Figure S9: ^{13}C-NMR Spectrum of compound (2d)

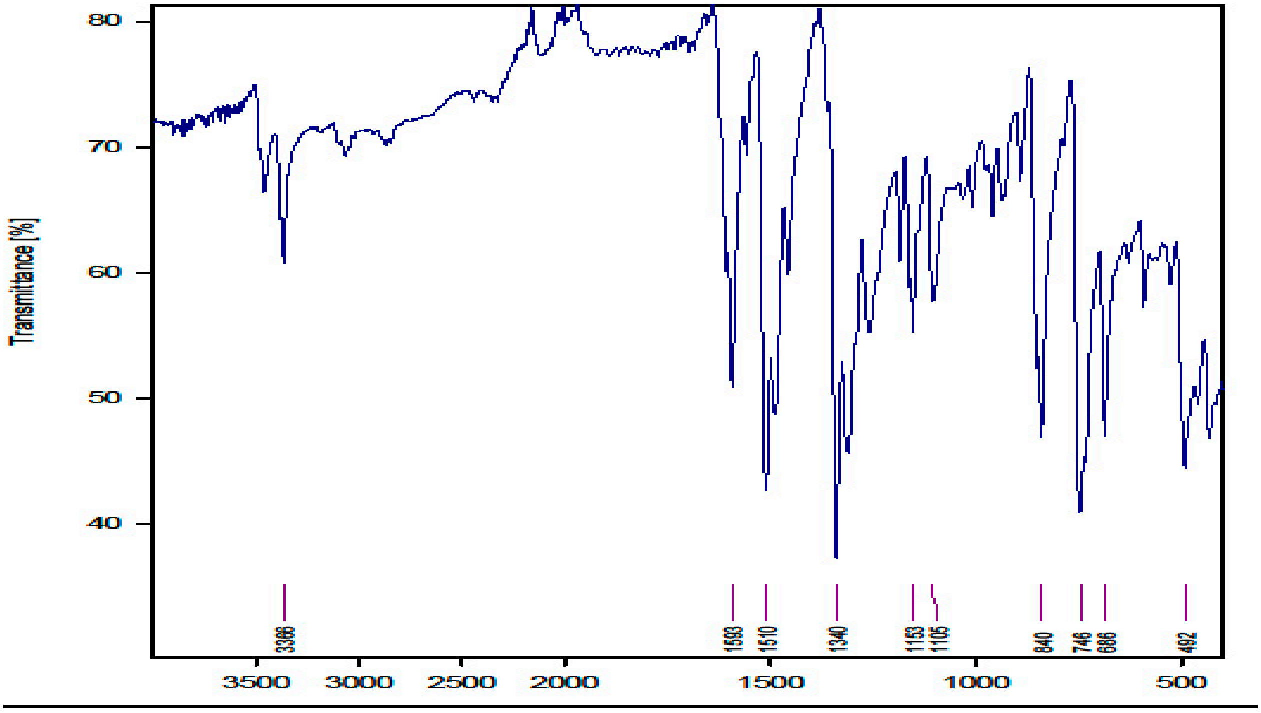

Figure S10: IR Spectrum of compound (2e)

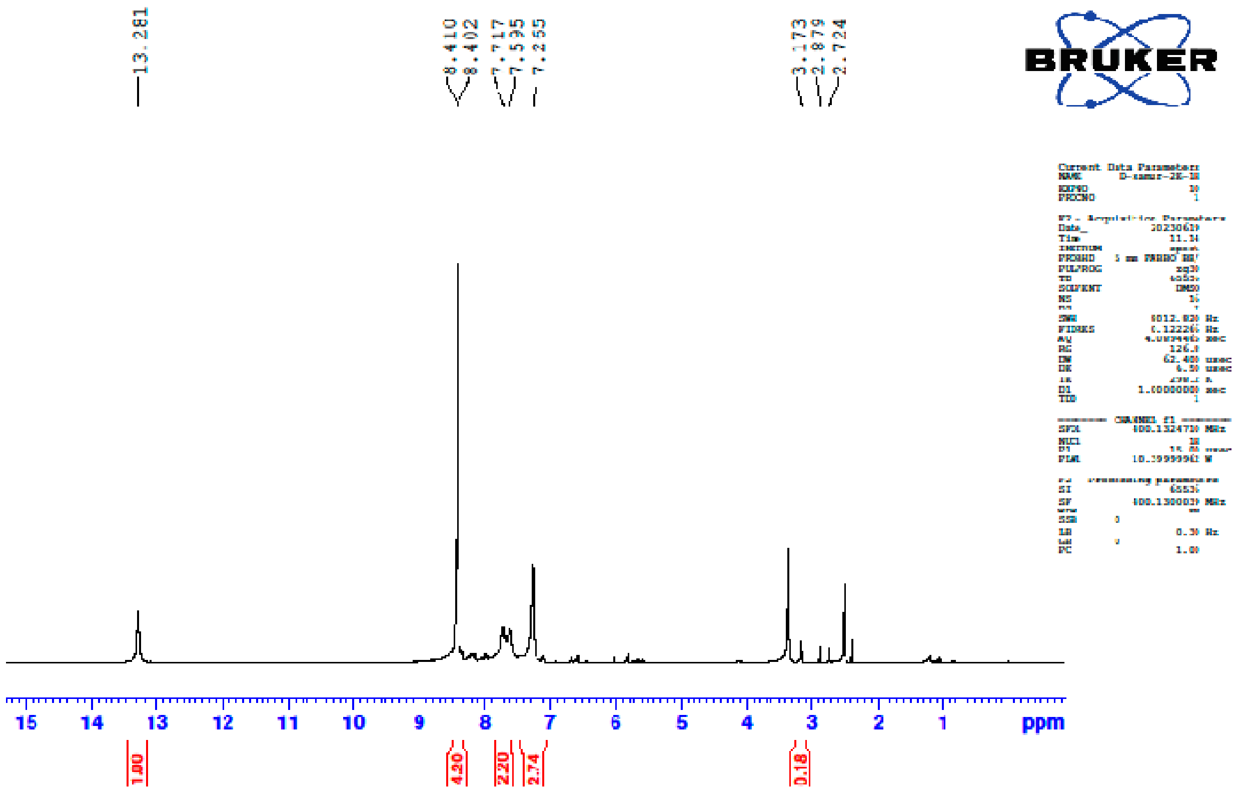

Figure S11: ^{1}H-NMR Spectrum of compound (2e)

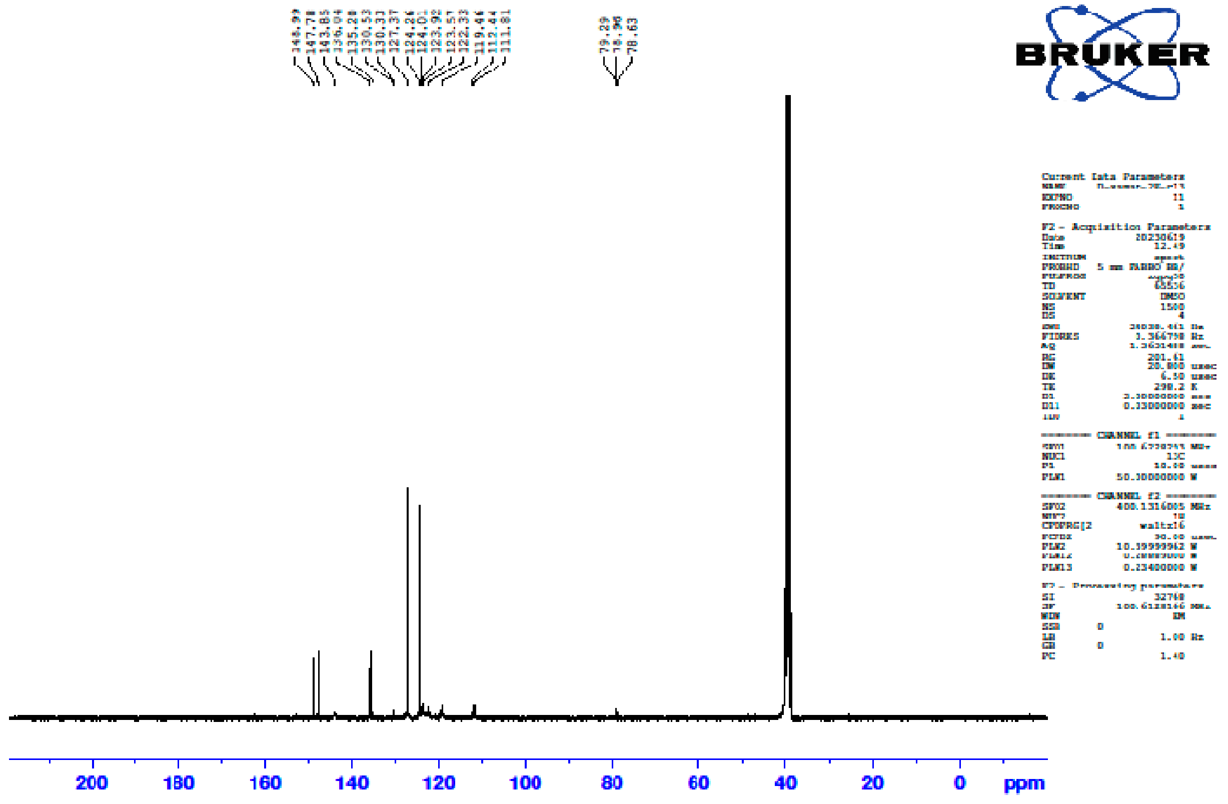

Figure S12: ^{13}C-NMR Spectrum of compound (2e

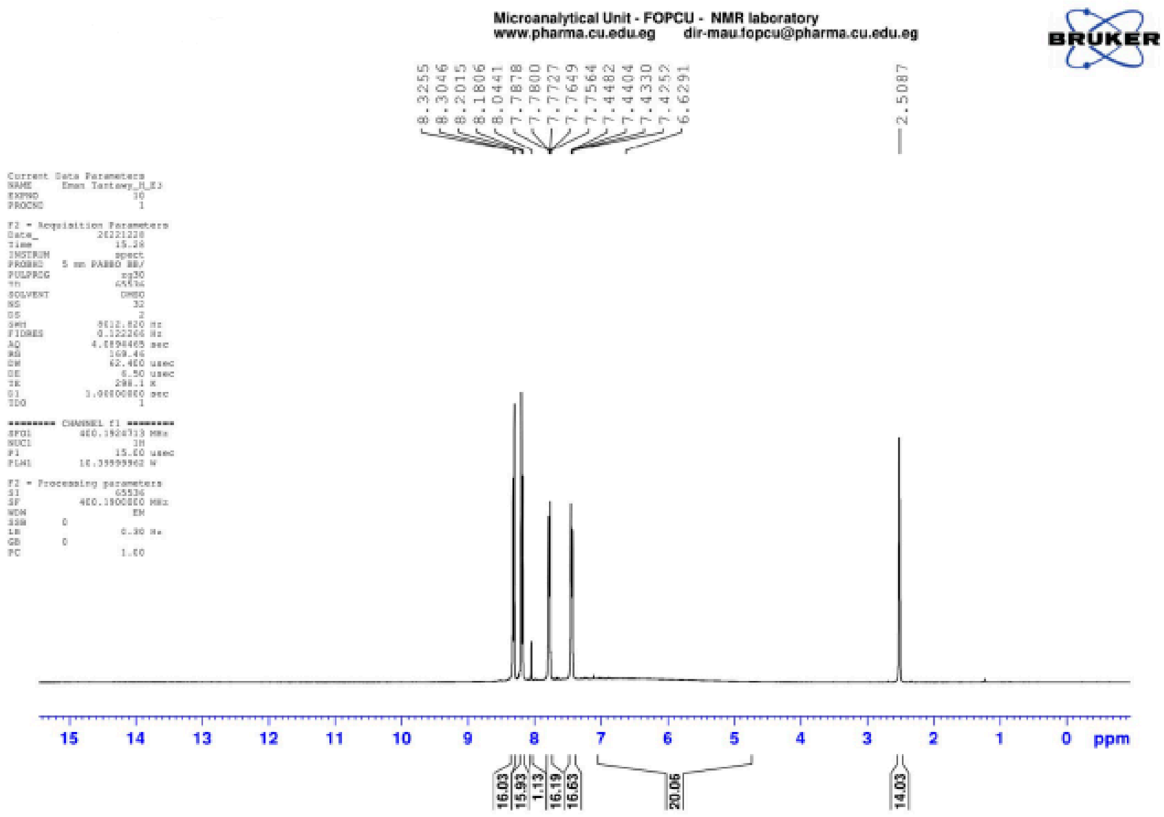

Figure S13: ^{1}H-NMR Spectrum of compound (2f) in D_2O

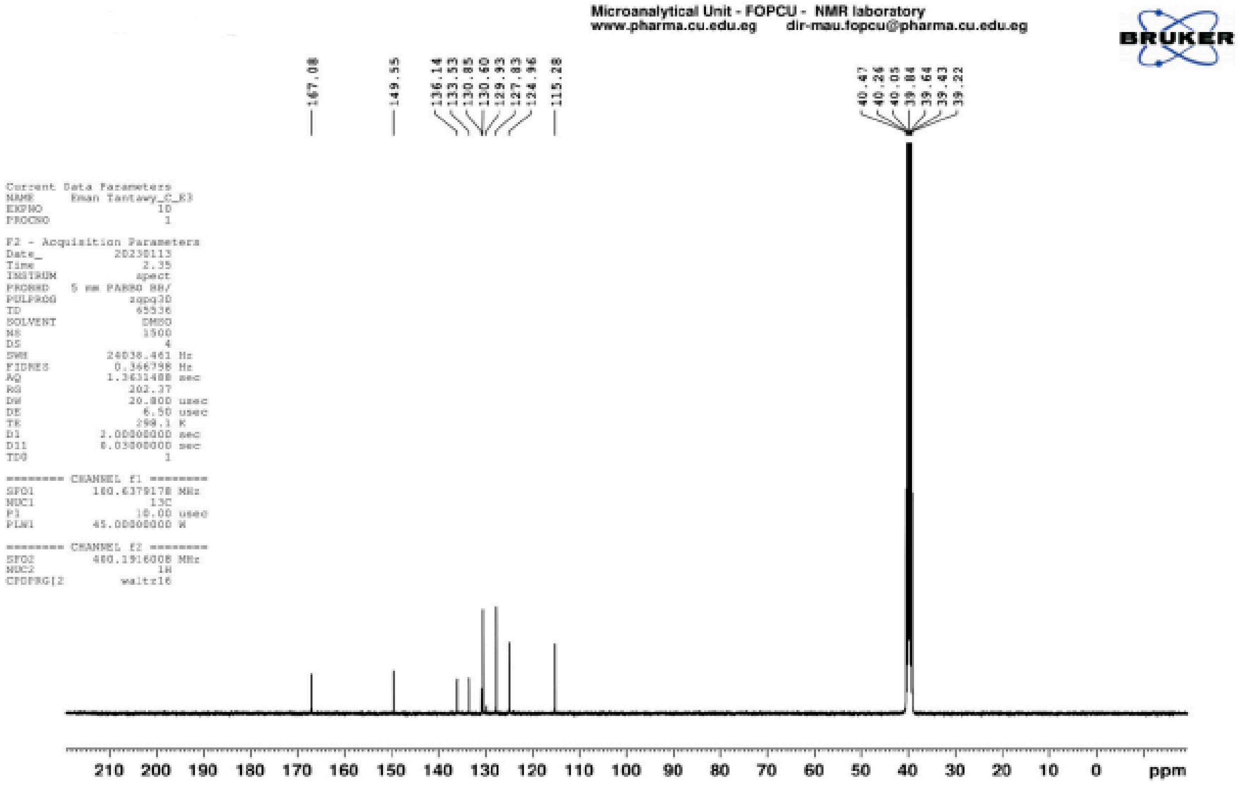

Figure S15: ^{13}C-NMR Spectrum of compound (2f)

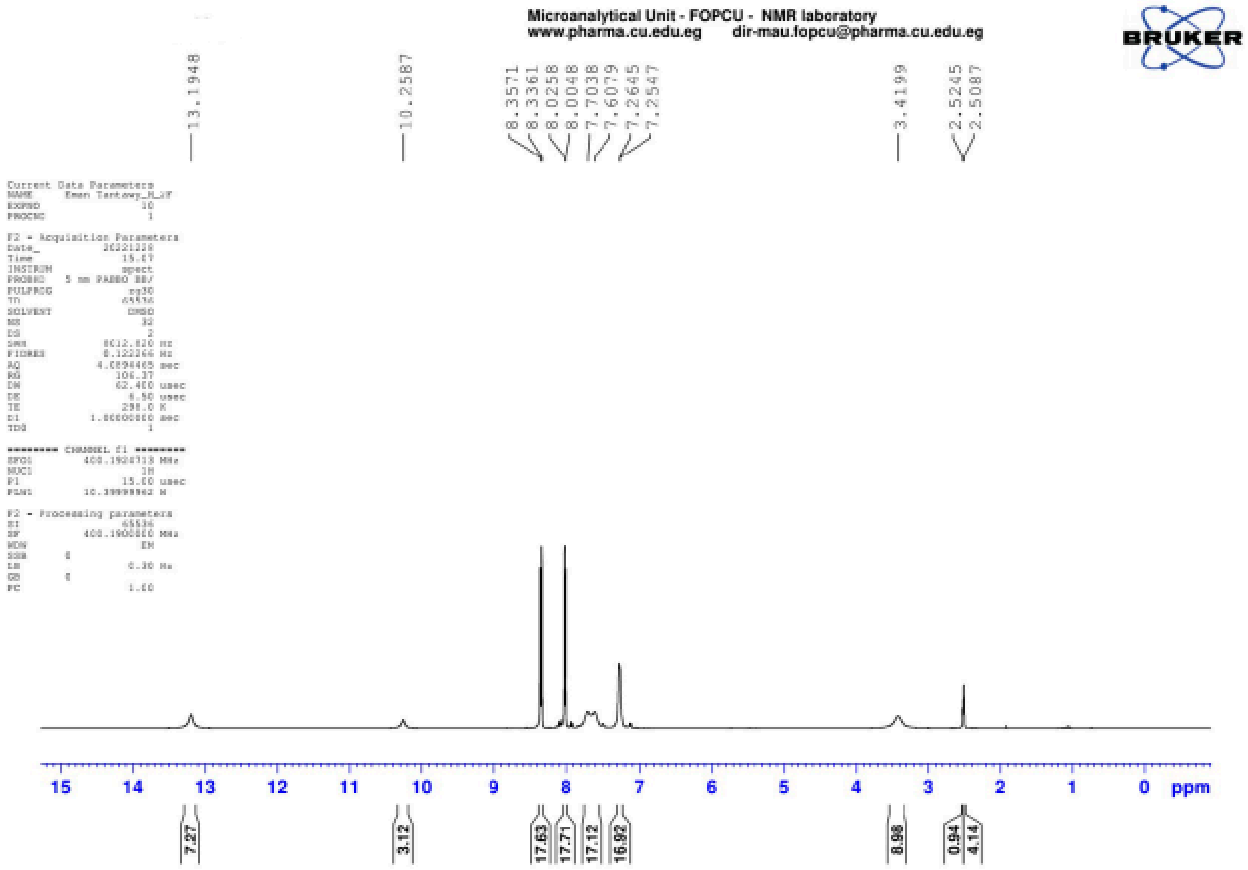

Figure S17: ^{1}H-NMR Spectrum of compound (3)

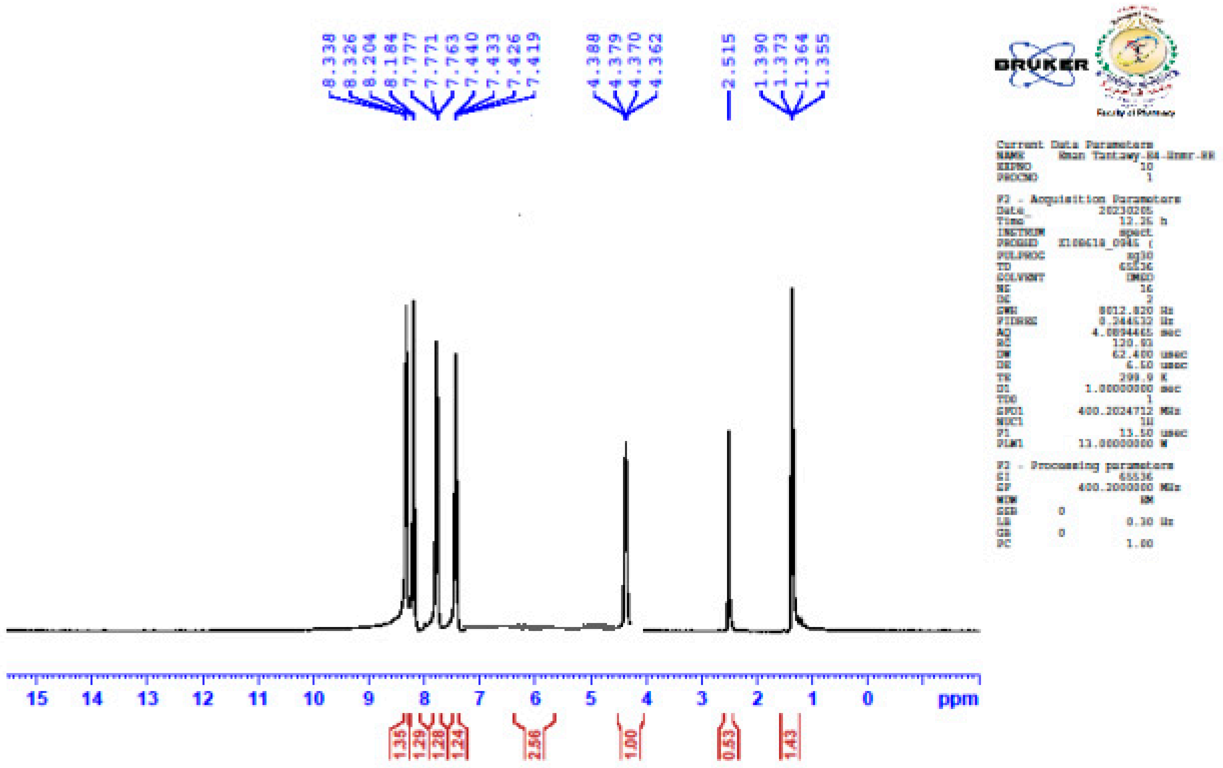

Figure S19: ^{1}H-NMR Spectrum of compound (4) in D_2O

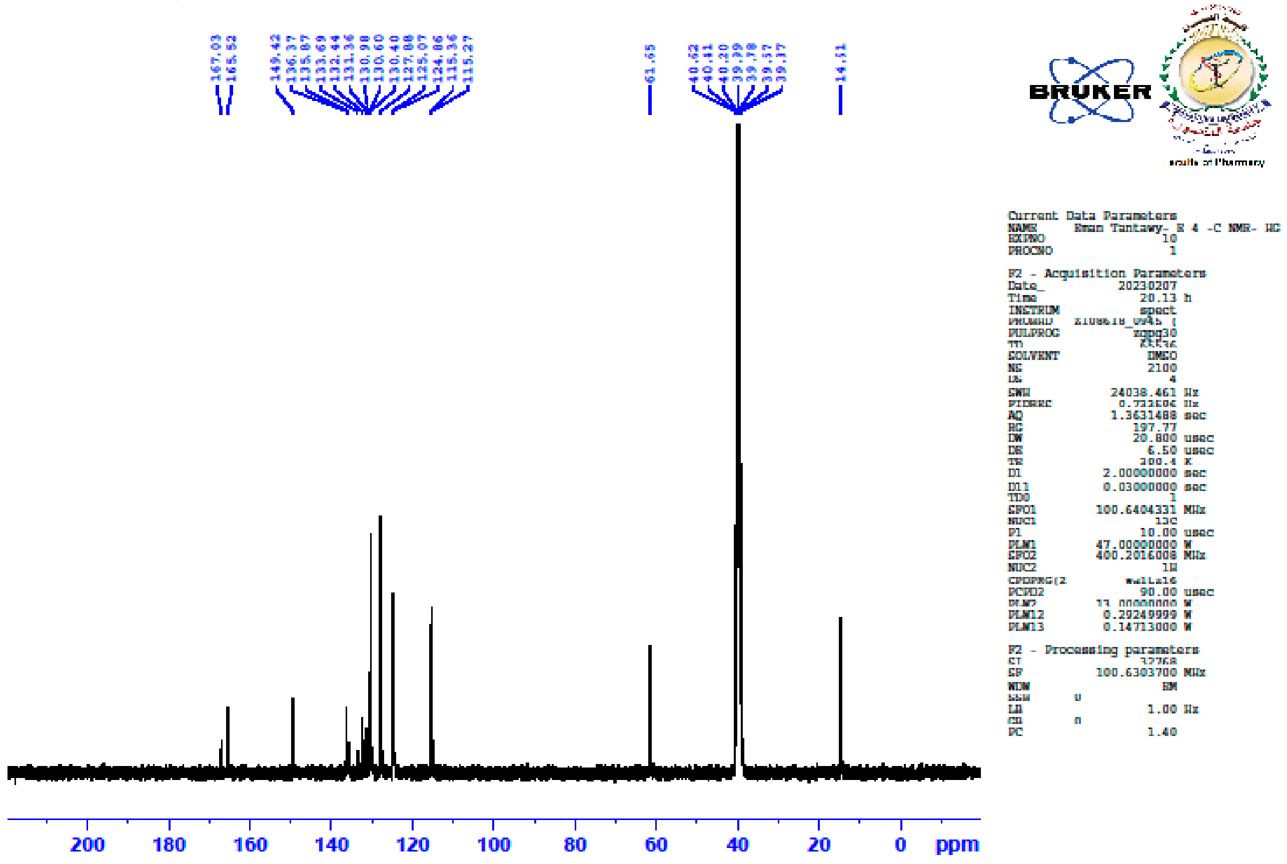

Figure S20: ^{13}C-NMR Spectrum of compound (4)

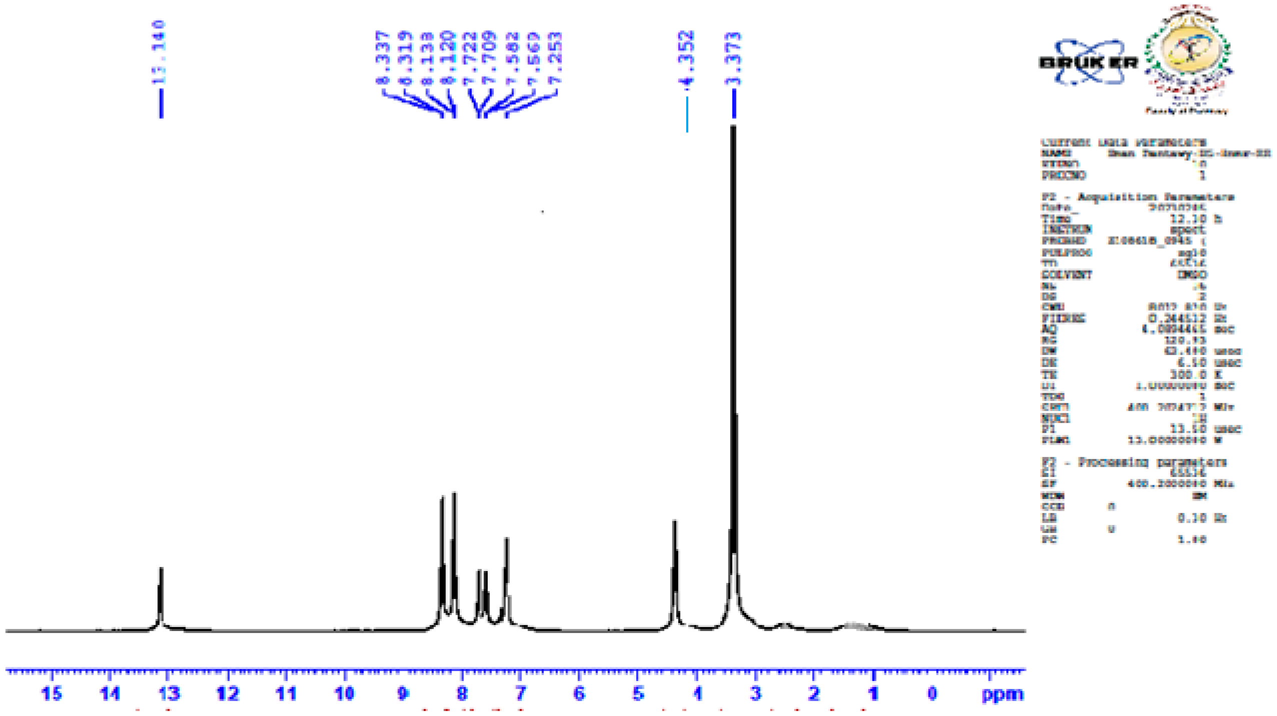

Figure S23: ^{1}H-NMR Spectrum of compound (5)

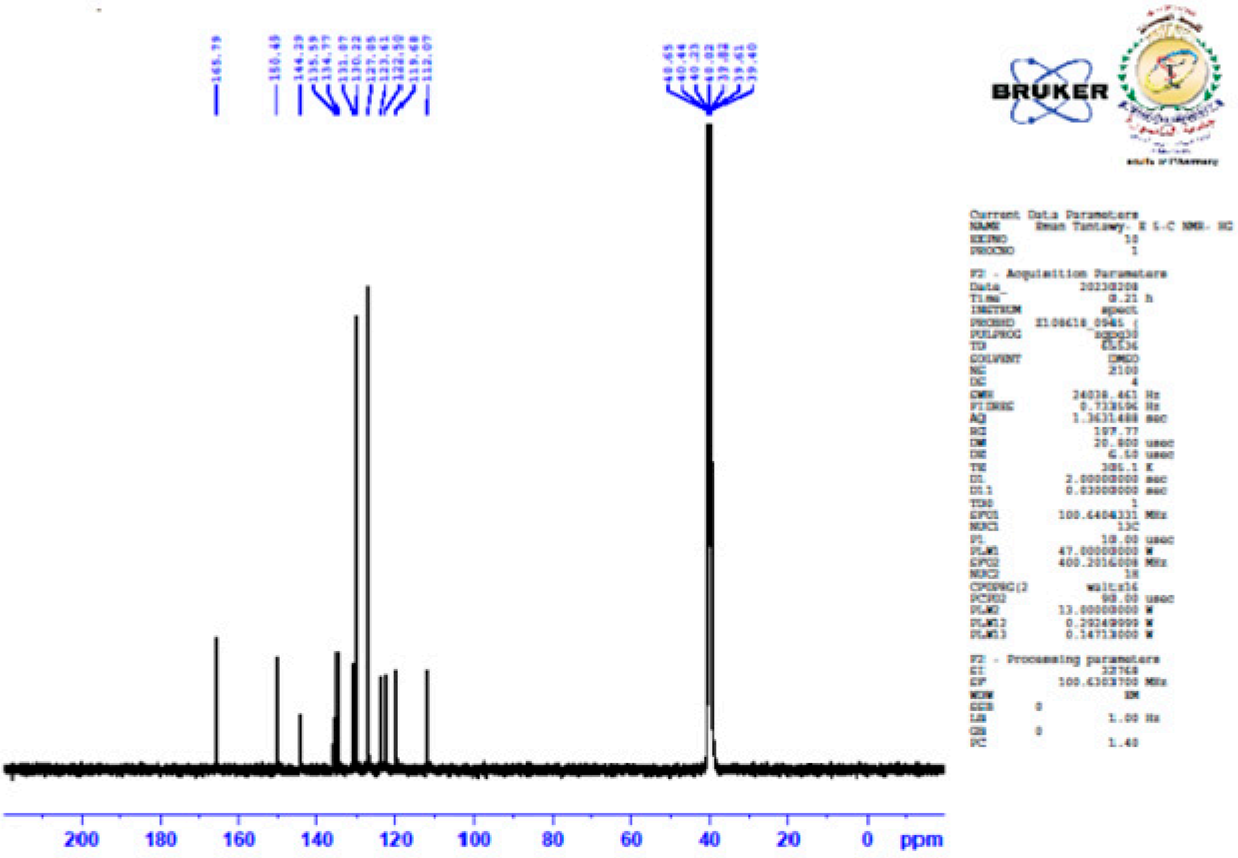

Figure S24: ^{13}C-NMR Spectrum of compound (5)
